# A Morality Based Target Audience Analysis and Quantum Open System Interaction Model for Propaganda

**DOI:** 10.3390/e28090987

**Published:** 2026-09-03

**Authors:** Mustafa Canan

**Affiliations:** Department of Information Sciences, Naval Postgraduate School, Monterey, CA 93940, USA; mustafa.canan@nps.edu

**Keywords:** disinformation, morality, propaganda, quantum open system, quantum cognition

## Abstract

Two people can view the same information but can take different actions. Most importantly, while thinking about moral and political issues, such as abortion, depending on the differences in the psychological systems, a perception of threat can arise. To exploit this aspect of the psychological systems, state and non-state actors make use of propaganda techniques to influence any target audience. Although the spread of disinformation is extensively studied in the information ecosystem, a model that represents the interactions between the schemata of the individuals and schemata of the perceptual stimuli has not been introduced. An interaction model is crucial to fathom the propaganda dynamics because while aiming to undermine the norms, values, and institutions in free societies, propaganda does not target the material objects or networks; it targets the psychological systems. Therefore, this research proposes to use the moral foundation theory (MFT) to define the system states of the interaction model for propaganda; doing so, the proposed model can represent the psychological systems of a target audience and schemata of the information stimuli with the same system states. This paper’s primary contribution is introducing an interaction model by capitalizing on a quantum open system model that captures the dynamics of two interacting systems in the information environment. The effects of non-selective measurement were studied by simulating a quantum open system-based cognitive model.

## 1. Introduction

People categorize social phenomena, actions, and other-than-themselves with their psychological systems. These psychological systems support diverse interpretative frameworks such that the same phenomenon can be perceived as praiseworthy by person A and blameworthy by person B. The story of a former member of the Westboro Baptist Church (WBC), Megan Phelps-Roper [1], can demonstrate the effects of the changes in these psychological systems. Her grandfather is the founding pastor, and her mother was the spokesperson of the church. She grew up in an environment in which the doctrine of the WBC permeated every aspect of her life [1]. She had been very articulate about the church and its exclusivist doctrine. When the church members decided to promulgate their doctrine via social media, Megan became the face of the church on Twitter. She argues that Twitter and other social media platforms enable the evolution of individuals and societies in unprecedented ways [1]. She shares her discussions (with then-husband-to-be) concerning notions such as commandments and truth. During those discussions, he was choosing his words in such a way that Megan would see the commandment and truth notions through the lenses of humility, gentleness, and compassion. Switching perspectives allowed her to interpret the very same notions through different lenses; in return, exclusivist interpretations have transformed into an inclusivist understanding. Although leaving WBC means estrangement from her family, Megan left.

Unfortunately, the changes in psychological systems via social media can lead to radicalism [2] or other social problems [3]. State and non-state actors actively shape the information sphere [4] and make use of propaganda techniques to encircle the information consumers in the information ecosystem [2,5,6,7,8,9,10]. These increased interactions involve the schemata of the individuals and schemata of the perceptual stimuli [11,12]. The state-of-the-art disinformation/fake-news/network studies, e.g., [13,14,15,16], do not address the interaction between perceptual stimuli and the cognitive dimension. While propaganda attempts to undermine the norms, values, and institutions in free societies, it does not target the material objects or networks; it targets the psychological systems that are utilized to perceive norms, values, and institutions.

This study develops a mathematical model of information processing that can capture the mechanism and dynamics of information processing in the context of propaganda or in general information processing with one set of cognitive state representation that can be used for the cognitive system and the information stimuli. The purpose of this study is to explicate via simulation how non-selective elicitation affects the cognitive system’s state evolution with respect to the case of not having non-selective elicitation.

## 2. Moral Foundation Theory

Two people can view the same information but can take different actions. For example, in the context of political issues such as abortion or same-sex marriage, with varying political preferences, the perception of threat can arise, and conflict can ensue. The problem here is not about being right or wrong; the problem is that individuals do not recognize the differences in the interpretative framework that are enabled while perceiving the same phenomena.

A book, movie, blog, speech, and tweet all have associated narratives. Narratives and frames are embedded in the story schemata, which describe the contextual regularities [12] of stimuli. When people interact with the regularities in the stimuli, their schemata are modified. A modified schema will affect the information-seeking behavior of the individuals, who then can be inadvertently directed to confirmation bias and trapped in a filter bubble. For example, to counter a conservative filter-bubble concerning abortion, the left should understand the interpretative framework of the conservative right. Thinking about moral issues, e.g., same-sex marriage, is different than thinking about what to do during a long weekend.

As a robust theoretical framework, MFT indicates that the human mind interprets social phenomena with five innate foundations: care/harm, fairness/cheating, loyalty/betrayal, authority/subversion, and purity/degradation; details are shown in Table 1 [17]. By using these five innate foundations, the degree of an individual’s political leanings can be expressed [18,19,20]. Haidt [20] argues that morality is innate and learned; morality results from adaptation processes that include psychological mechanisms. As a result, morality can be used to represent the schemata of a target audience and the schemata of the information stream with the same parameters.

**Figure 1:**
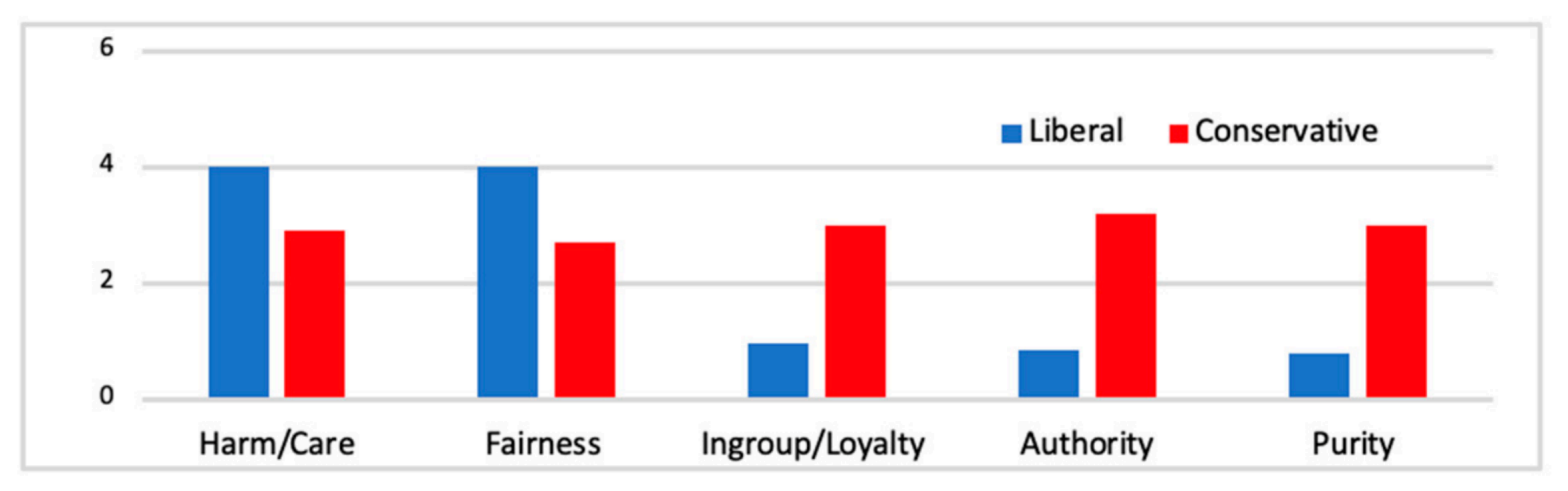
The five pillars of morality for two major political preferences.

**Figure 2:**
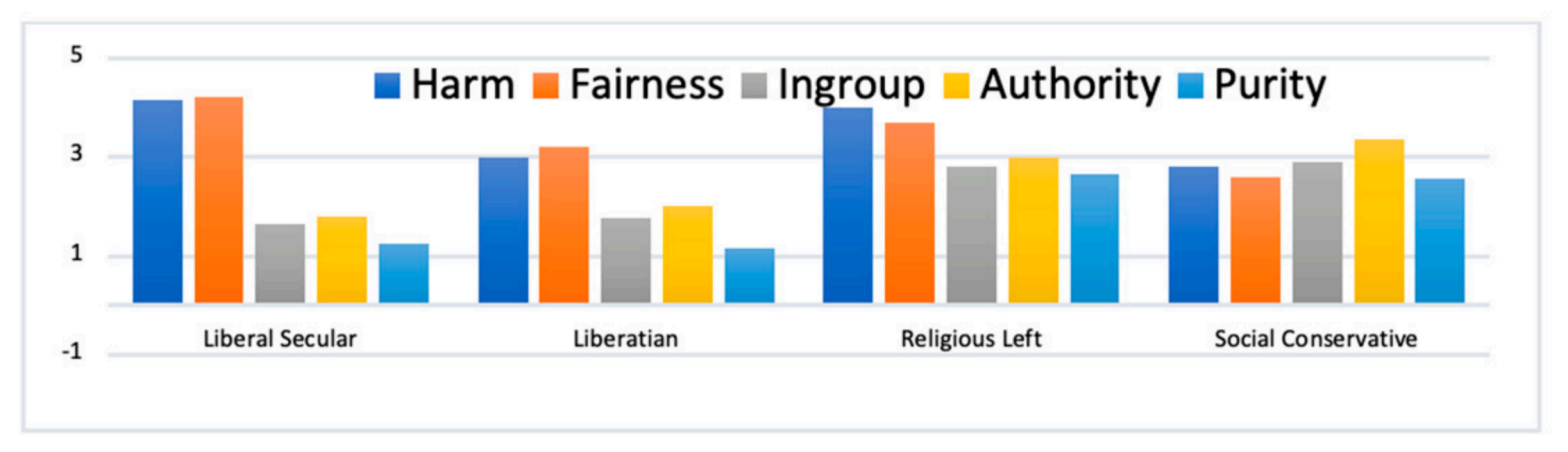
Moral foundation patterns for four clusters.

The five pillars of morality for two major political preferences exhibit different moral matrices [17] Figure 1. The moral matrix of the left is dominated by two of the five moral foundations; the right, social conservatives, have an equally weighted moral matrix [20]. Subsequent MFT studies introduced four categories concerning the patterns in moral pillars [21] Figure 2. The two new categories (liberation and religious left in Figure 2) elucidate the state of the moral mind of the individuals, who are typically categorized as moderates or bi-conceptual [22].

Although morality is innate, it is highly dependent on environmental influences [17,23]. MFT elucidates a framework in which one can study how the human mind is prepared to learn/adapt values and norms that are related to a diverse set of social problems [23]. MFT has commonalities with the construction of social reality [24]. For example, the left perceives the legalization of same-sex marriage as a way to reduce harm for the innocent victims and to increase fairness for everyone [21]; the right perceives same-sex marriage as a different cultural practice, an action against the authority of the church and an impure action [21]. Therefore, the two sides cannot fathom each other unless they comprehend the differences in their interpretative framework. The MFT can provide a unified framework to study the interaction between the human mind and perceptual stimuli in the information environment.

An important product of the MFT is its dictionary [25,26,27]; the first MFT dictionary [28] had a limited number of words. Although the first dictionary had been used as the theoretical foundation for several voting prediction studies [21,29,30,31,32] and provided robust results, a newer MFT study [27] suggested a newer version of the dictionary [25], shown in Table 2, which is more comprehensive. MFT delineates characteristics of moral development, such as the construction of social reality, contextual learning/adaptation, being highly intuitive, and being dependent on environmental influences.

### Propaganda and Moral Foundation Theory

MFT can ameliorate the models of information processing/in the information environment that include disinformation propaganda. In this work, four aspects of propaganda that connote the characteristics of the moral foundation theory are discussed.

The first aspect: Morality is a product of the construction of social reality. Information connotes the reality [33] and includes disinformation [34]; as a result, taking the totality of information [35] into account is critical to capture the full threat vector of disinformation propaganda. For example, while focusing on the dissemination of disinformation and fake news or critical nodes in social networks, e.g., [13,14,15,16], the interactions that occur between perceptual stimuli and the cognitive dimension can be unheeded. These interactions are part of the construction of social reality, which delineates the perceptual adaptation processes, such as X becomes Y in context C [24]. Since disinformation propaganda aims to undermine the norms, values, and institutions in free societies, it targets the process of construction of reality; in return, cognitive representations of norms, values, and institutions change, and the magnitude of change should be accounted for in the threat vector of disinformation propaganda.

The second aspect: Contextual learning and adaptation. Propaganda exploits cognitive peripheral routes, such as watching TV while doing other work, or skimming through updates in social media feeds [8,9,10] so that it can increase its interaction with the target audience [7]. These interactions involve the schemata of the individuals and regularities of the perceptual stimuli [11,12]. As a result, the changing schema of the individual constrains the action and perception of the individual, such as defining the other-than-self [36,37]. In the current information ecosystem, the reciprocal relationship between the user and the environment is augmented with more contextual effects [38]. This provides a perfect ontology for disinforming [38]; as a result, the freedom of choice is controlled, culture is directed, and the danger of being systemically disinformed becomes very real [11].

The third aspect: Morality is highly intuitive and influenced by the environment. Propaganda attempts to smear a specific interpretative framework for the concepts that are pertinent to social norms, values, and institutions [16,38,39]; in doing so, it can induce conflict and confusion concerning social issues. Propaganda standardizes ideas, reinforces prevailing stereotypes, and provides patterns of thought concerning all the aspects of social life. In doing so, propaganda attempts to codify social, political, and moral standards [6]. The distracting elements of the information ecosystem enact fast thinking; hence, the intuitive system controls the decisions [3,20], framing effects increase [40], and analytical reasoning can be hindered [20,22,41].

The fourth aspect: Propaganda cannot create something out of nothing; it must attach itself to a sentiment, an idea that exists in society [6]; it must be associated with a foundation that already exists in the minds of the constituents of the target audience. Propaganda must consist of fundamental psycho-sociological bases, such as pillars of MFT, because propaganda that conflicts with the accepted structure of a target audience would have no possibility of success [6].

## 3. Human Mind and Stimuli Interaction

The overlapping concept for propaganda, influence, social networks, disinformation, misinformation, and information warfare research is the human element. The models in this field mostly capitalized on the theoretical construct of classical probability theory (CPT). For example, a Bayesian model in this context is a monotonic accumulation model [42] with the constraints of the Bayesian model that has the property of the conjugate update [43]. Belief update in the Bayesian model in the context of propaganda/disinformation/influence operations is sequential and order-independent [43,44,45,46]. Modeling influence with a Bayesian approach not only ignores the order of the information received but also shows no sensitivity between having information one-by-one vs. receiving all at once [45]. Markov models of information sharing/opinion update/influence [47] can also be considered adaptive; however, there is no time sensitivity in the Markov models. A Markov model of influence follows an ergodic behavior and monotonically converges to a stationary distribution [43,48]. A pure quantum probability model (QPT) based model [49,50] can lose the coherence of the system transiently with intended interactions; for example, a decision-making entity elicits a judgment or a decision [50,51,52,53], or by gradually adapting to the environment [48,53]. Both the Markov and Bayesian modeling approaches aligned with the older propaganda/disinformation/influence [54] efforts in which the volume of information was not inflated and, hence, the target audience would monotonically absorb the evidence with the cues in the information stream. For contemporary propaganda/influence operation modeling, these modeling approaches fail to capture ambiguity/indeterminacy [55,56]. More importantly, Bayesian- or Markov-based models represent uncertainty in a way that it decreases as the evidence accumulates; if this is the case, which may not be all the time, the increase in ambiguity/confusion with more information cannot be captured by these CPT based models [44,53,55,56,57]. In the pure quantum model case, the problem is continuous oscillation because it does not consider the interaction element as a continuous dynamic, hence demonstrating indefinite oscillation.

The modern-age propaganda is like a firehose [54]. Modern information/influence/propaganda operations are high-volume, repetitive, engagement-driven, and aim at inducing confusion along with persuasion [58,59]. Consequently, the new information ecosystem, in which humans are drawn into information, becomes the perfect habitat for these operations. People in this ecosystem may not know what to pay attention to. Information in the new ecosystems is too timely, which may entail spontaneous action [38]. Its repetitive, continuous stream with high volume of information generates an environment in which humans have very little time to think between perceptual stimuli [54,60,61,62]. This environment gives rise to fast thinking situations in which framing effects can increase [40]. In this environment, humans are exposed to an excessive amount of information, and hence the analytical reasoning processes can be hindered [20]. For example, a conflict can easily arise if the interlocutors use incompatible frames to understand a social phenomenon [22]. Therefore, a framework that can represent destabilization and engagement-timing that can account for judgement/choice/decision elicitation can capture features of modern-age information warfare.

Hitherto research indicates that although QPT based models capture some of the shortcomings of CPT based models, using a pure quantum model also introduces modeling shortcomings. However, the quantum open system (QOS) modeling combines Markov and pure quantum models in one equation with single continuous probability distribution [43,48,51,52,53].

### 3.1. Quantum Open System Modeling

QPT is recognized as a comprehensive method in cognitive modeling [44,49,57]. Mathematical axioms of QPT ameliorate the puzzling experimental findings in cognitive science as they did for physics [63]. One of the peculiar contributions of the QPT is that it can capture different types of uncertainty [64], which may be characterized as either epistemic or ontic [48,51,53,56]. Epistemic uncertainty has to do with a lack of information and can be minimized through gathering additional information from the environment. Ontic uncertainty describes the uncertainty or indeterminacy experienced due to the superposition of the cognitive states that are based on possible outcomes [44]. Ontic uncertainty, indeterminacy can only be resolved through interaction with the environment, for example, eliciting (including non-selective measurement [65]) a category/judgment/decision. The distinctions between epistemic and ontic uncertainty are important for modeling propaganda, especially the ontic type of uncertainty that can allow one to address ambiguity/confusion that is internal to the target audience [51,53,56,61].

Depending on the situational constraints, quantum-like and classical dynamics interchangeably dominate human information processing. To represent the change in dynamics continuously with one single probability distribution, quantum open system (QOS) [66] has been adapted for use in decision modeling [48,51,53]. QOS can capture both the non-commutative relations between incongruent perspectives and interaction effects while also including classical Markovian dynamics. Furthermore, QOS can relate the dissipation and adaptation dynamics of the decision-making with a single probability distribution as a function of time [52].

#### 3.1.1. QOS Equation

The quantum open system is an extension of the Gorini-Kossakowski-Sudarshan-Lindblad (GKSL) equation (please see Appendix B and Appendix C for detailed derivation and proofs), also known as the Lindblad equation [51,67,68], shown in Equation (1).

(1) $$\frac{d}{dt}\rho \left(t\right)=-i\cdot \left(1-q\right)\cdot \left[H,\rho \right]+q\cdot L\left(\rho \right)$$

(2) $$L\left(\rho \right)=\sum \gamma_{ij}\cdot \left(L_{ij}\cdot \rho \cdot L_{ij}^{†}-\frac{1}{2}\left\{L_{ij}^{†}\cdot L_{ij,},\rho \right\}\right)$$

(3) $$\left\{\left(L_{ij}^{†}\cdot L_{ij,}\right),\rho \right\}=\left(L_{ij}^{†}\cdot L_{ij,}\right)\cdot \rho +\rho \cdot \left(L_{ij}^{†}\cdot L_{ij,}\right)$$

(4) $$\left[H,\rho \right]=H\cdot \rho -\rho \cdot H$$

The Equation (1) is the QOS equation and is used to describe cognition and human decision making [48,51,53,69]. The first part of Equation (2) $i\left[H,\rho \right]$), represents the quantum component. The second part of the Equation (1), L(ρ)), represents the classical Markov component. The weighting parameter, q, in Equation (1) provides a means to weight which element (e.g., Markov or quantum) will dominate the system. For example, when q=0, it signifies that the system is in a fully quantum regime, which indicates a higher ontic uncertainty based on the model simulation; when q=1, quantum dynamics no longer take place, and the system model becomes Markovian. The quantum open system model begins with oscillatory behavior, and due to the interaction with the environment, the oscillation dissipates to a stationary distribution due to the classical dynamics, such as Markov models [67].

The challenge with using Equation (1) as it was used in earlier applications in evidence accumulation [48,51], and trust [53] is that it does not work as it is for the target audience analysis. The reason is that evidence accumulation and trust models in [48,51,53] are both one-dimensional quantum/Markov walks. The modeling assumption in these approaches is like a spatial quantum walk in which states are linearly ordered. If the same assumption is used for MFT pillars, the resulting order will be Care→Fairness→Loyalty→Authority→Purity. However, this is just an arbitrary order that can be found in the seminal MFT work [20,23]. This assumption would introduce a nearest-neighbor structure, which means that with this arbitrary order, some of the moral foundation pillars, cognitively, are assumed to be adjacent, resulting in a chain sequence behavior.

#### 3.1.2. Building Hamiltonian

The general Hamiltonian equation is:

(5) $$H=\frac{\pi }{2}\cdot \left[c\cdot C+V_{0}\cdot diag\left(s\right)\right]$$

In Equation (5) c(=35) [48] represents the coupling constant, C is the coupling matrix to determine the off-diagonal terms of the Hamiltonian, and s represents the information stream that determines the diagonal potential based on the MFT pillars of the information stream. $V_{0}(=500)$ [48] represents the potential depth, and $\frac{c}{V_{0}}$ (for a detail discussion for these two parameters please see Appendix D) determines the dynamics between adjacent pillars: transition vs. information stream strength. These two modeling parameters are modeling constants and do not approximate any human cognitive characteristics. The use of s as the potential of the attack Hamiltonian rests on the assumption that the relative frequency of moral framings in the information stream translates into the relative strength of the bias those framings attempt to impose on the audience’s cognitive system. This assumption is consistent with findings on the amplified diffusion and priming effects of moral-emotional language in social media in earlier studies. However, it should be noted that word frequency characterizes the content broadcast, not the content received/processed by a user or, in general, a target audience; hence its validation belongs to the future empirical study as discussed in Section 6. The numerical value choices for c and $V_{0}$ are based on the 35 and 500, which are taken from [48]. In the case of chain approach, the coupling matrix will be:

(6) $$C_{chain}=\left(\begin{array}{ccccc} 0 & 1 & 0 & 0 & 0\\ 1 & 0 & 1 & 0 & 0\\ 0 & 1 & 0 & 1 & 0\\ 0 & 0 & 1 & 0 & 1\\ 0 & 0 & 0 & 1 & 0 \end{array}\right)$$

The chain sequence approach was the first Hamiltonian matrix built in this work. However, the $C_{chain}$ in Equation (6) introduces an arbitrary adjacent coupling to the MFT pillars. In Equation (6), each column represents one MFT pillar (from left to right: Care→Fairness→Loyalty→Authority→Purity), and the off-diagonal 1 entries represent the transition possibility.

A theoretically correct approach is to restructure the coupling matrix, C, in the Hamiltonian by using block foundations of the MFT pillars [17,19]. There are two blocks for MFT pillars, which are the individualizing foundation and the binding foundation. The individualizing foundation includes two pillars, which are Care and Fairness; the binding foundation includes three pillars, which are Loyalty, Authority, and Purity. With this approach, the coupling matrix becomes:

(7) $$C_{block}=\left(\begin{array}{ccccc} 0 & 1 & v & v & v\\ 1 & 0 & v & v & v\\ v & v & 0 & 1 & 0\\ v & v & 1 & 0 & 1\\ v & v & 0 & 1 & 0 \end{array}\right)$$

In Equation (7) v represents the strength of coupling between the pillars in a block. For example, Care and Fairness in the individual foundation block coupled with a strength of v. This coupling strength v is between 0 and 1. When v=0, the coupling constant in Equation (7) becomes $C_{no\text{-}coupling}$; if v=1, the coupling between the pillars becomes fully coupled. This means that every pillar couples directly with the other pillars. However, there is no evidence in the MFT literature to support this super-coupling approach. Having a v value between 0 and 1 provides a coupling within the blocks of MFT.

#### 3.1.3. Building Markov Intensity Matrix

Building the Markov component of the QOS equation is similar to building the Hamiltonian. Choosing the Markov intensity matrix $K_{ij}$ without considering the individual/binding block constraint would mean that the classical component of the QOS results in alignment with the information stream schema quickly and under all conditions. In the model, the rate of arrival is expressed by λ; as a result, without the clustering constraint, the rate of change is:

(8) $$\lambda \cdot s_{i}$$

where $s_{i}$ in Equation (8) is the information stream. There are options for the information stream profiles for IRA; one is based on the words in the corpus and the other is based on the hashtags, which are shown in Equations (9) and (10) respectively. The details of this discussion are presented in Appendix A.

(9) $$s_{profile\_words}=\left[0.30\;0.15\;0.18\;0.15\;0.22\right]$$

(10) $$s_{profile\_hashtags}=\left[0.08\;0.12\;0.30\;0.25\;0.25\right]$$

By using the same block constraint used in Equations (6) and (7), the new intensity matrix will be:

(11) $$K_{ij}=\lambda \cdot C_{ij}\cdot s_{i}$$

Since $C_{block}$ in Equation (6) is symmetric, $C_{ij}=C_{ji}$, $K_{ij}\cdot s_{j}=K_{ji}\cdot s_{i}$, K·s=0 is ensured and the stationary distribution will be still $s_{i}$.

#### 3.1.4. Operationalizing K and H Matrices in QOS

The QOS equation shown in Equation (1) requires adjustment to operationalize the K and H matrices. The density matrix, ρ, is a 5 × 5 matrix that represents the general cognitive system of the target audience with the pillars of the MFT. The diagonal entries $p_{i}$ represent the probability of a foundation pillar based on the target audience MFT category that is shown in Figure 1. In this work, a new super operator that acts on ρ is introduced to account for the defense mechanics of the target audience in the same equation. This new operator $L_{total}$ is (please see Appendix B for details):

(12) $$\frac{\partial \rho }{\partial t}=L_{total}\cdot \rho$$

The super operator $L_{total}$ is written as:

(13) $$L_{total}=\underset{\begin{array}{c} \mathrm{attack}\\ \mathrm{weight} \end{array}}{\underbrace{\gamma }}\cdot \left[\underset{\begin{array}{c} \mathrm{Quantum}\\ \mathrm{dynamics} \end{array}}{\underbrace{q\cdot H}}+\underset{\begin{array}{c} \mathrm{Maikov}\;\mathrm{attack}\\ \mathrm{dynamics} \end{array}}{\underbrace{\left(1-q\right)\cdot D_{attack}}}\right]+\underset{\begin{array}{c} \mathrm{Defense}\\ \mathrm{Markov} \end{array}}{\underbrace{\overset{\begin{array}{c} \mathrm{defense}\\ \mathrm{weight} \end{array}}{\overbrace{\left(1-\gamma \right)}}\cdot D_{defense}}}$$

In Equation (13) there are two forces in action, which are attack and defense. The attack force dynamics can be quantum and classical Markov dynamics. Each of these dynamics represents the operators that can act on the cognitive system by considering the information stimuli. Quantum dynamics in the attack component in Equation (13) is similar to the fast thinking aspect, which is also known as system 1 [70], which derives the confusion when exposed to cues in an information stream. On the other hand, the Markov dynamics in the attack component in Equation (13) is akin to a more analytical slow-thinking system 2 [70]. The defense component in the super-operator $L_{total}$ only includes classical Markov because of the assumption that defense mechanics requires more analytical reasoning and it will be slower, as assumed in system 2 [70]. The attack weight γ is between 0 and 1; the quantum vs. classical weight q is between 0 and 1 [48,51,53,69].

The hitherto Hamiltonian discussion in this work provides a sufficient construct to build the model; however, the K requires more discussion to distinguish the $K_{attack}$ and $K_{defense}$.

$K_{attack}$ Intensity Matrix

In Equation (13) by following the discussion in [48,51,53,69], $D_{attack}$ of the attack component can be written as:

(14) $$D_{attack}\left(\rho \right)=\sum_{k\neq j}K_{kj}\cdot \left(L_{kj}\cdot \rho \cdot L_{kj}^{†}-\frac{1}{2}\left\{L_{kj}^{†}L_{kj},\rho \right\}\right)$$

The intensity matrix $K_{attack}$ in Equation (14) is calculated as follows:

(15) $$K_{ij}=\left\{\begin{array}{cc} \lambda {\cdot C}_{ij}\cdot s_{i} & i\neq j\\ -\sum_{k\neq j}\lambda \cdot C_{kj}\cdot s_{k} & i=j \end{array}\right\}$$

Based on Equation (15), the off-diagonal elements are scaled by $C_{ij}$, hence, the transition within a moral cluster (individual/binding) can occur at full rate because $C_{ij}=1$. On the other hand, cross-cluster block transition rates will be weighted by v, where $C_{ij}=v$.

The intensity matrix of the attack component $K_{attack}\;$ is a 5 × 5 Markov intensity matrix with a stationary distribution $s_{i}$, the chosen information stream. The state of this 5 × 5 Markov matrix is based on the pillars of the MFT. The attack dissipator in Equation (14) $D_{attack}$ represents either analytical reasoning that derives accepting the information stream schema; or it represents the adaptation to the information environment. The diagonal elements of D(ρ) reproduce the classical master equation, which is $\frac{dp}{dt}=K\cdot p$, where p is a probability column vector. Including $K_{ij}$ in Equation (14) delivers the consistency of the dissipator factor to operate on all of the elements of the density matrix, not just the diagonal elements.

$K_{defense}$ Intensity Matrix

The defense intensity matrix is built in the same way as $K_{attack}$ in terms of the cluster block matrix. The only difference between $K_{attack}$ and $K_{defense}$ is that the stationary distribution in the defense intensity matrix is represented as $p_{0}$, which is the prior distribution of the audience’s cognitive system based on the MFT pillars.

(16) $$K_{ij}=\left\{\begin{array}{cc} \lambda {\cdot C}_{ij}\cdot p_{0} & i\neq j\\ -\sum_{k\neq j}\lambda \cdot C_{kj}\cdot p_{0} & i=j \end{array}\right\}$$

By using $K_{defense}$ in Equation (16), the dissipater, $D_{defense}$, is built as in Equation (14).

## 4. Simulation Analysis and Results

The goal of the analysis is to use the QOS model to simulate the evolution of the cognitive system represented by ρ under different conditions that are characterized by q and γ values to understand the behavior of the target audience under different conditions. The parameter values that are used in this analysis are shown in Table 3. The text analysis, Appendix A, provides the quantitative characterization of the attack through $s_{i}$; the literature provides the characterization of the defenders and the prior distribution; and the simulation combines the two components in this work.

The choice of these parameters is to analyze the interaction between a target audience with information stream at boundary conditions, e.g., fully quantum, minimal attack, and decision elicitation. There are two conditions in each analysis: control and treatment. A treatment condition in this simulation represents a group that, in an experiment, would be the group that is prompted to elicit a decision, choice, or judgment. The treatment condition represents the elicitation, which is a categorization of a phenomenon or a cue. The control condition in this simulation represents a group that would decide without eliciting a categorization or an intermediate judgment. The comparison between the control and treatment conditions via simulation is done to explicate how a categorization/intermediate judgment can affect the evolution of the cognitive state of a target audience. This state evolution is an important feature of the quantum models because prompting a confused target audience can be directed to a chosen outcome [61,71], if the amplitude of the confusion is tracked. When a target audience is exposed to an information stream for a long time, the dissipater dynamic dominates and off-diagonal terms of the density matrix become zero [53]. The logic of this elicitation is to replicate this adaptation process with forced categorization. The coherence is destroyed (ρ→diag(diag(ρ)), but the population is untouched. The elicitation acts on coherence (not on a single system state, which is one of the MFT pillars) such that an induced ambiguity can be used as an advantage to substantiate a firm understanding at the cognitive level. This process is also known as non-selective measurement [65]. An elicitation event, in the present interpretation, is any interaction situation that prompts a user to commit (even if it is inadvertently) to a moral framing of the cued phenomenon. This can be through participating in a poll, a reply or quote tweet in response to a solicitation, deciding whether to share or endorse, or reacting to content. The measurement is non-selective in nature because the model does not condition on which framing the user choose/commits; instead, the elicited ensemble is averaged over outcomes, corresponding to a population where an engagement is prompted but individual responses are not tracked. This is an interpretive correspondence, not an empirically established correspondence. The dataset records the tweet contents, not the interaction histories of exposed users; hence, its validation is part of the future empirical study.

Thus, the elicitation is the act of categorization itself, averaged over all possible answers weighted by their probabilities. No single foundation is picked out; the whole superposition is collapsed into a classical mixture across all five pillars, with the population weights preserved.

From the propaganda perspective, elicitation is the moment a propaganda feed prompts a reaction; for instance, these prompts can be

- share this,
- which side are you on,
- react now,
- who would you vote for in November.

The target audience is pushed to substantiate a moral framing of the cue; for example, is this cue a:

- Loyalty matter?
- Care matter?
- Authority matter?

To sum up, the act of measurement in simulation captures the treatment condition; in the quantum model, the act of being forced to categorize a cue affects the ambiguity that is captured by the oscillation [61]. In addition to the parameters listed in Table 4, the initial cognitive state distribution of each group, two idealized, ensemble-level audience profiles derived from published moral foundations survey research; the scope and limits of this two-profile design are stated in Section 3. The two idealized $p_{0}$ distributions are as follows:

(17) $$p_{0\;Conservative}=\left[0.20\;0.20\;0.20\;0.20\;0.20\right]$$

(18) $$p_{0\;progressive}=\left[0.35\;0.35\;0.12\;0.10\;0.08\right]$$

### 4.1. Pure Quantum Case #1 q=1, γ=1

**Figure 3:**
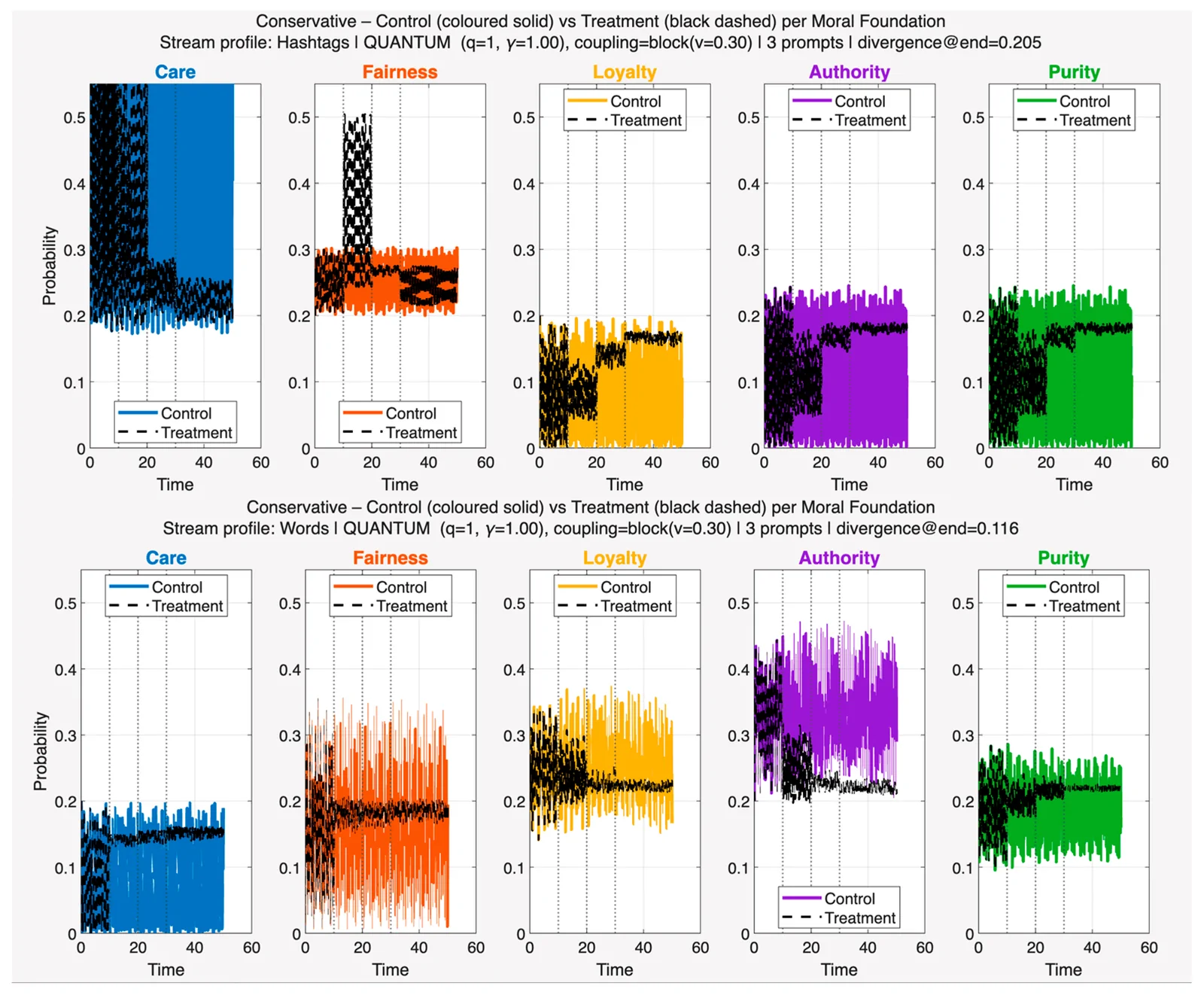
Conservative Probability distribution per Moral foundation. When the information stream is set to hashtag, the individual experiences more confusion compared to the word information stream. As can be seen in Equations (9) and (10), the word information stream is more aligned to the conservative distribution.

**Figure 4:**
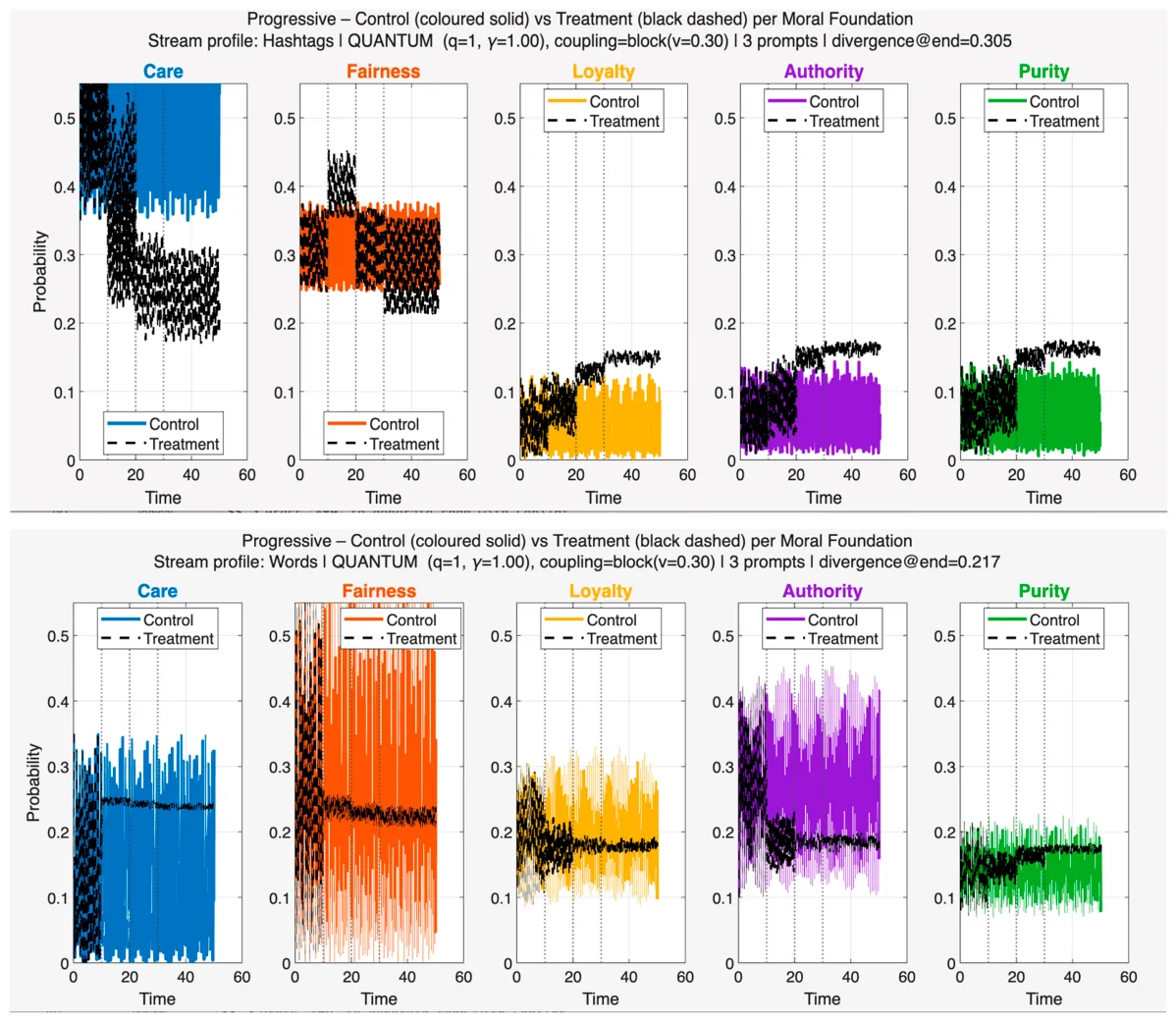
Progressive Probability distribution per Moral foundation. When the information stream is set to hashtag, the individual experiences less confusion compared to the word information stream. As can be seen in Equations (9) and (10), the word information stream is more aligned to the conservative distribution; as a result, a progressive perspective experiences confusion.

In this condition, the behavior of the target audience was simulated for both of the information streams that are shown in Equations (9) and (10). Since q=1, in the attack component there is no Markov contribution and there is no defense mechanics involved because γ=1. Under these two conditions, the behavior of a target audience over time is dominated with full quantum dynamics. For the control condition in Figure 3 and Figure 4, both the conservative and progressive audiences demonstrate oscillating behavior in the model when they are exposed to the information streams that have different distributions. This oscillation represents the ontic type of uncertainty [48,51,53,56,61] that represents the confusion an individual in the target audience experiences due to the superposition/coherence of the cognitive states. Ontic type uncertainty describes the internal ambiguity that an individual experiences while processing any new information [53,55,56,60,61].

**Figure 5:**
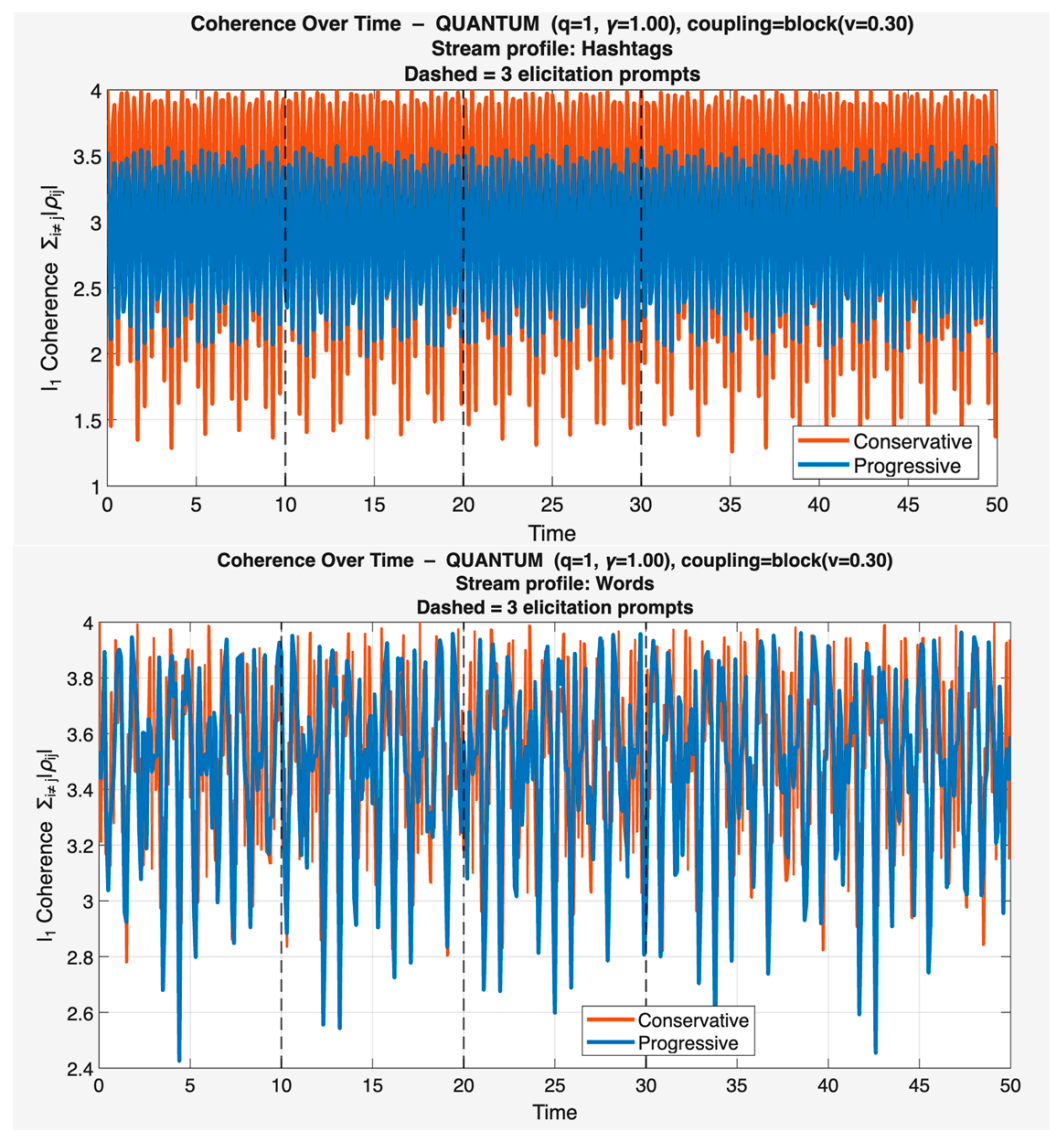
Coherence over time for (no treatment).

In this condition, there is no dissipating dynamics; as a result, until an elicitation is prompted for a categorization/decision, the coherence is not interrupted, as shown in Figure 5. The treatment condition simulates when an audience is prompted to elicit a categorization through a non-selective measurement process; within the model, it demonstrates different behavior than the control condition. The graphs in Figure 3 and Figure 4 demonstrate, in the model’s dynamics, that the introduced block coupling to avoid chain progress captures the progressive audience resistance to the binding stream, shown in Equation (10) (system stays in Care/Fairness domain). However, repeated elicitation and non-selective measurement manage to force some probability across the moral-family boundary into the binding foundations (Authority, Loyalty and Purity). This supports the idea that the individualizing/binding boundary introduces distinct vulnerabilities, and prompting is the mechanism that makes the breach possible.

The sociological significance of the transition between foundations in the simulation analysis is worth elucidating. In MFT, the binding foundations, which are Loyalty, Authority, and Purity, are the parochial foundations [20,21]. The binding foundations are associated with group binding behavior that holds members together and draws a boundary that defines who is not in the group [23]. Empirical work in moral and political psychology has linked the binding foundations to out-group derogation, prejudicial attitudes, and support for exclusionary policy [18,21,23], and Purity-based framing has been associated with dehumanizing characterizations of out-groups [18,21,23,27,28]. The result of the analyzed Twitter data in Appendix A supports these framings. For example, the anti-Islamic and anti-immigrant content documented in Table A1 is constructed as Loyalty appeals with betrayal narratives and in-group solidarity. Purity appeals, for example, with an invasion metaphor. The elicitation-driven shift of probability across the individualizing/binding boundary in Figure 3 and Figure 4 represents, in the context of this theoretical work, how propaganda can weaponize/exploit/create boundary-drawing situations. It should be noted that the limit of this interpretation is as follows. Heightened binding-foundation does not constitute a discriminatory attitude, and the introduced coupling parameter v is a constant parameter in the model. As a result, this parameter is not an empirically tested/adjusted threshold of attitude change. What the introduced model identifies is a dynamic that describes susceptibility to us-vs.-them context that can be bolstered by the repeated elicitation. The comparison and testing between simulated state trajectories and measured attitudes remains an empirical question, addressed in the future-work research in Section 6.

For a conservative target audience, the prior distribution, $p_{0}$ shown in Equation (17), is uniform; as a result, a simulated conservative population experiences more oscillation compared with the progressive audience, a comparison shown in Figure 5. As can be seen in Figure 3, the breach between the two blocks is higher in Fairness with non-selective measurement after prompting to elicit. This is something that needs to be investigated further because of the nature of the quantum random walk that considers spatial priority (closer to the binding block) to Fairness.

**Figure 6:**
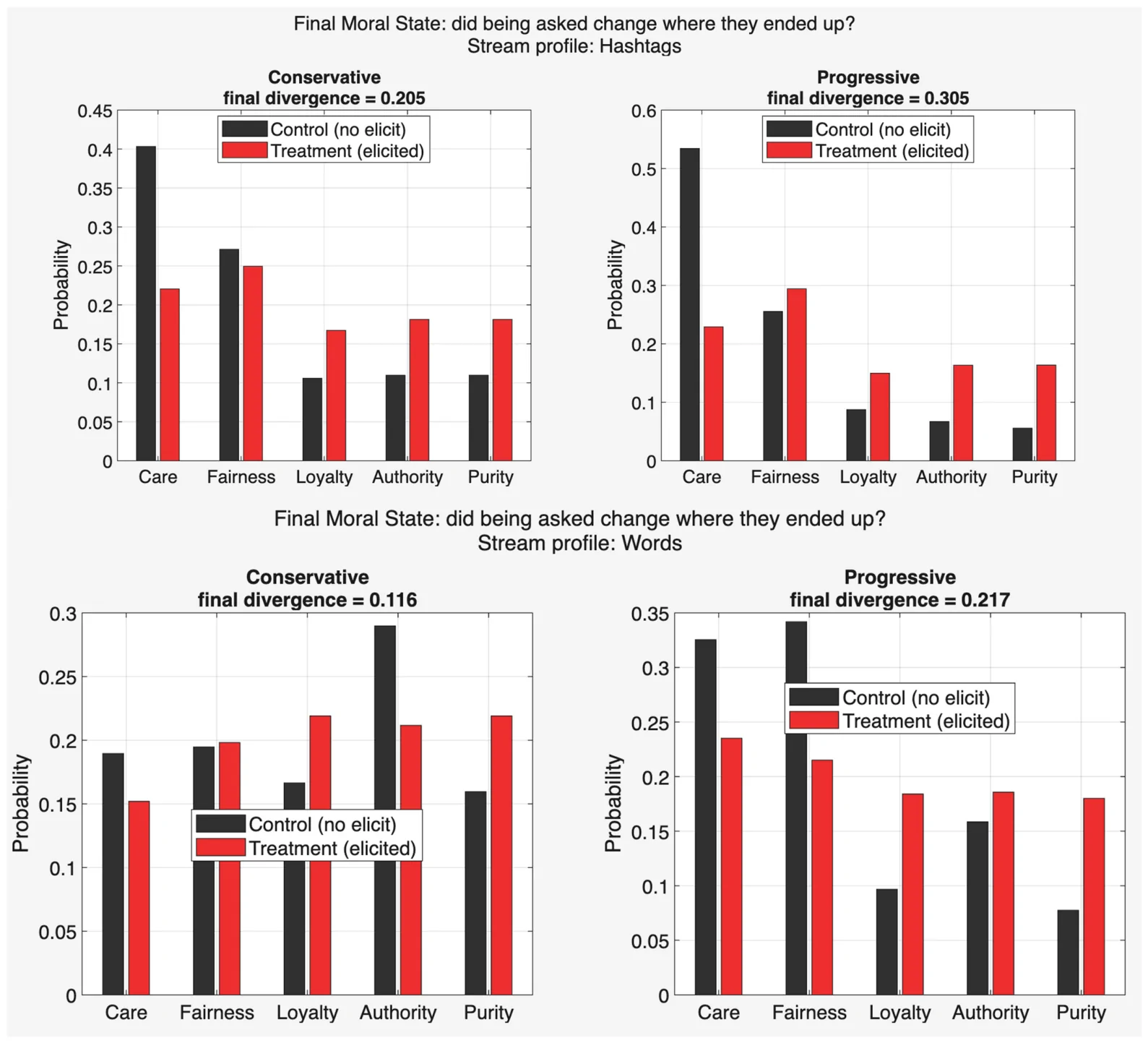
Final Moral Foundation Pillar Distribution for full quantum case, full attack, no defense.

Figure 6 shows the final moral foundation probability distribution per pillar. The progressive audience in Figure 6 has become more aligned with the elicitation process. This simulation model indicates that although the information stream induces confusion, the elicitation determines the final non-kinetic effect. As discussed in [54], confusion is the goal for modern-age propaganda; however, substantiating an alignment between the information stream and the cognitive system is required to have an effect. For the simulated conservative target audience, eliciting also helps to align with the information stream MFT distribution.

**Figure 7:**
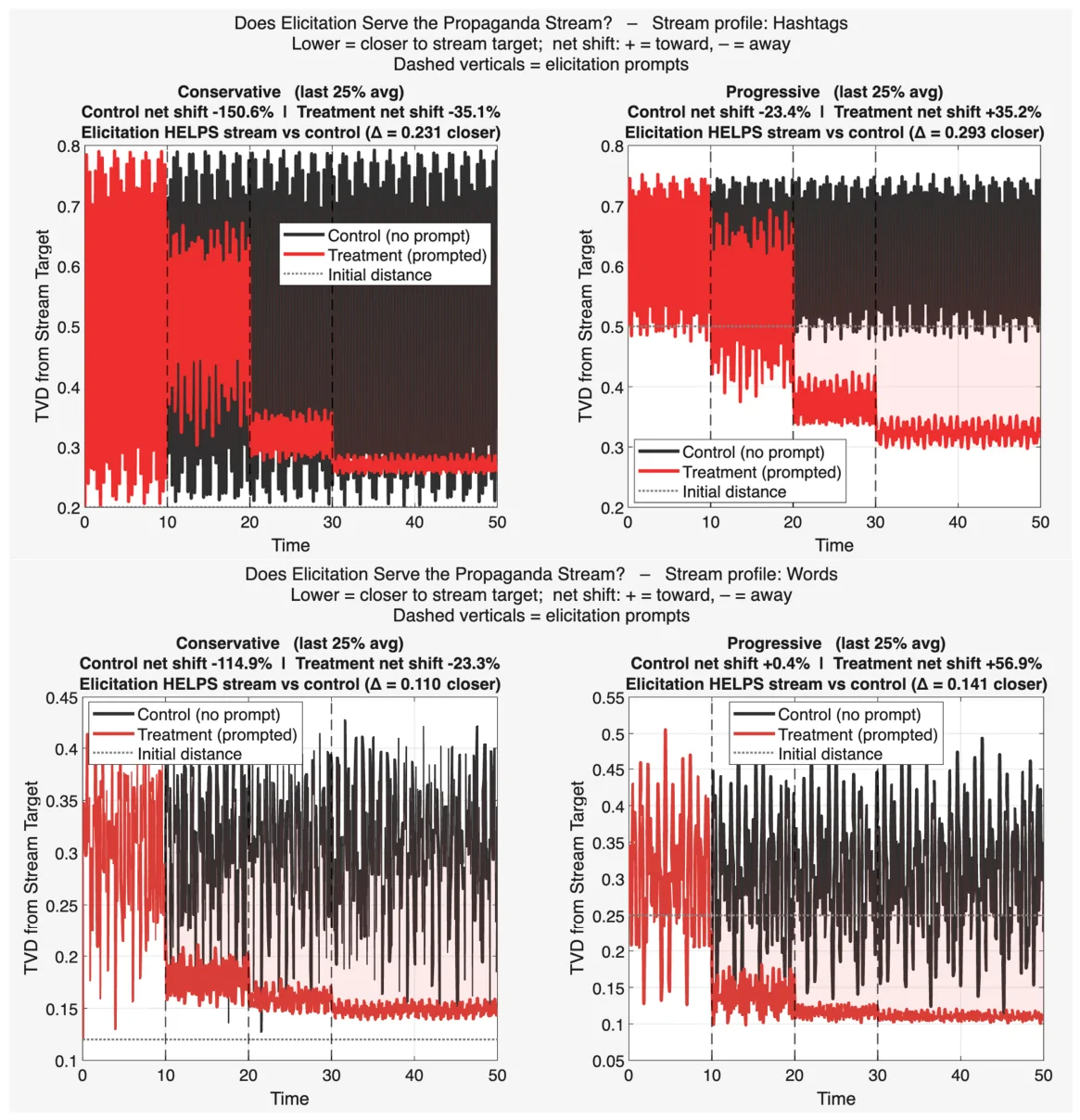
The total variation distance (TVD) between the target audience’s initial moral state ($p_{0}$) and the propaganda stream (s). The elicitation helps under both conditions in favor of the information stream. As a result of the interaction, the target audience adapts the perspective that is disseminated by the information stream.

Figure 7 demonstrates how eliciting dynamics in the model improves the non-kinetic effects of propaganda. The total variation distance is calculated as:

(19) $$TVD\left(p,s\right)=\frac{1}{2}\sum_{i=1}^{n}\left|p_{i}-s_{i}\right|$$

Both the conservative and progressive target audiences were affected by the information stream and got closer to the stream distribution when elicitation, non-selective measurement, occurs.

### 4.2. Pure Quantum Case #2 q=1, γ=0.01

**Figure 8:**
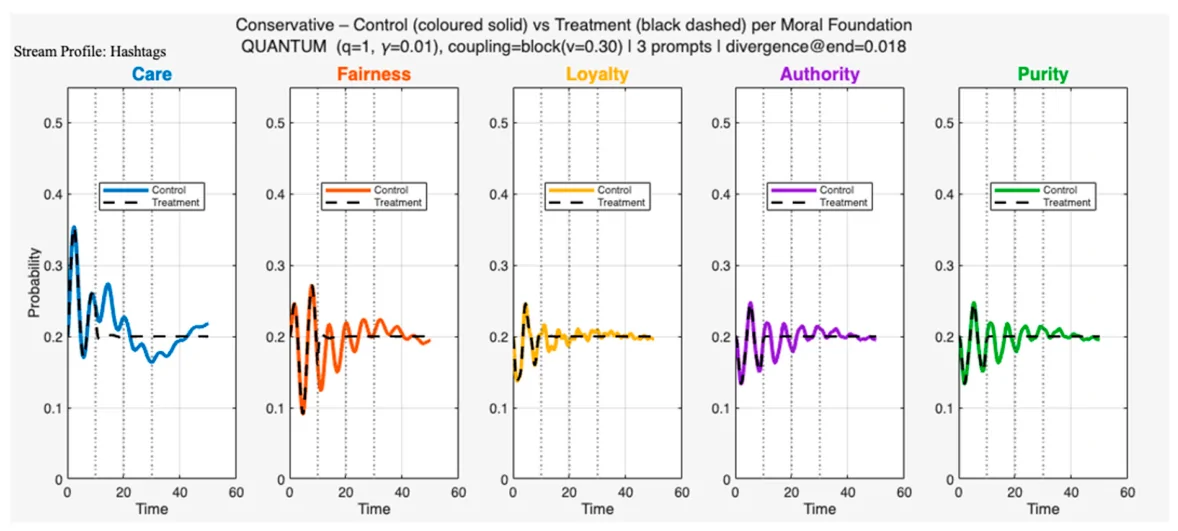

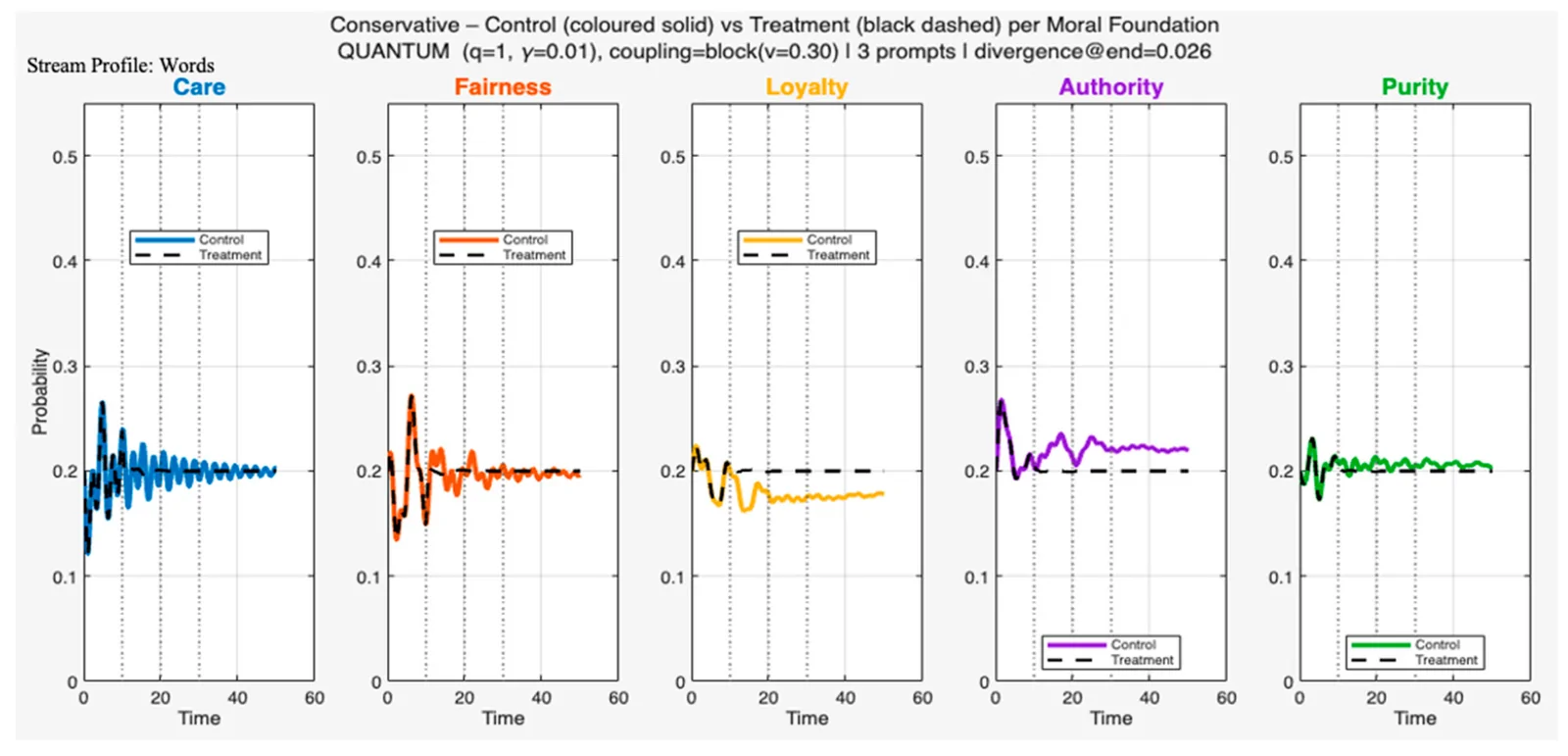
Conservative Probability Distribution per Moral foundation pillar full quantum heavy defense (γ=0.01).

**Figure 9:**
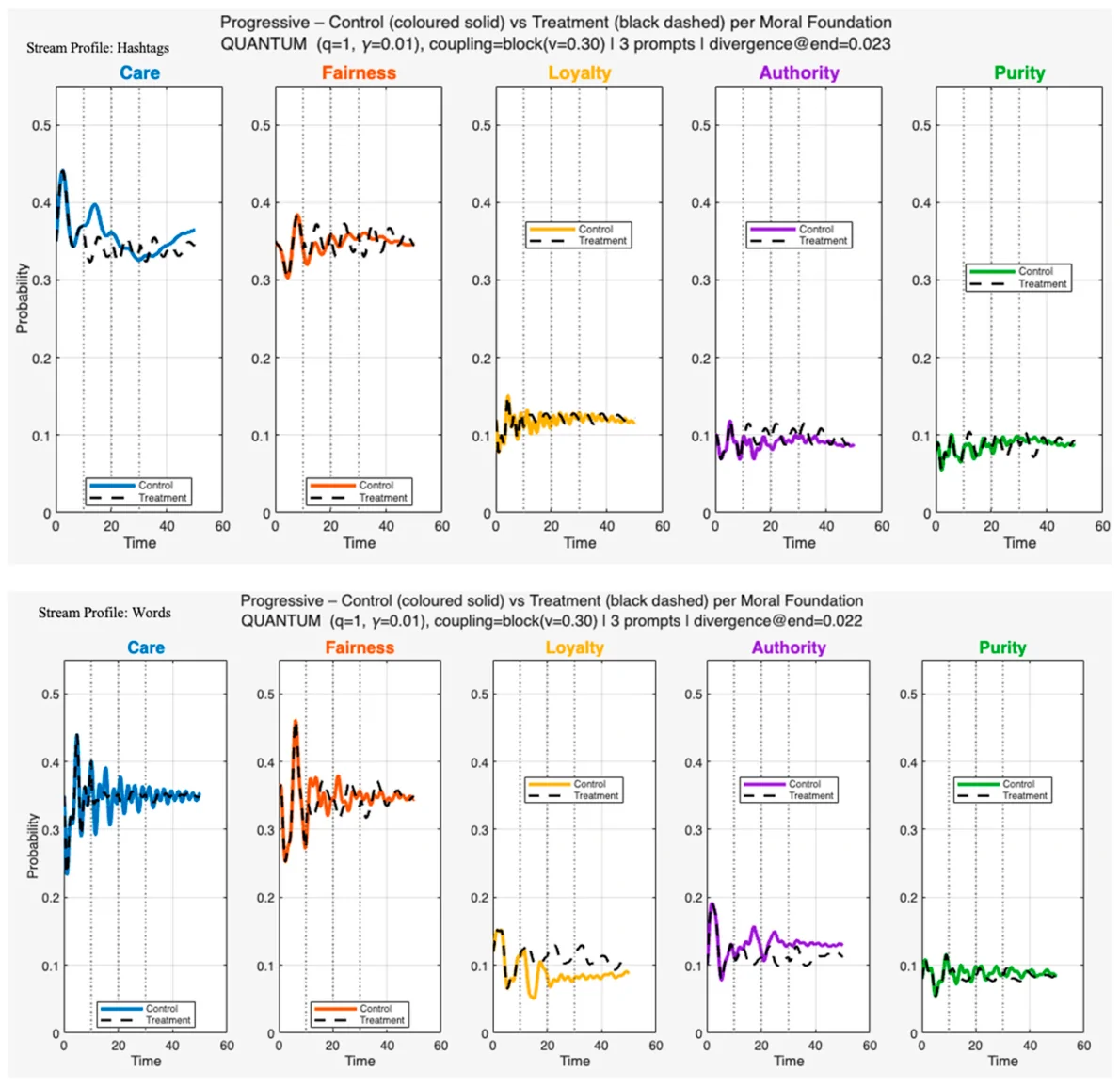
Progressive Probability Distribution per Moral foundation pillar full quantum heavy defense (γ=0.01).

**Figure 10:**
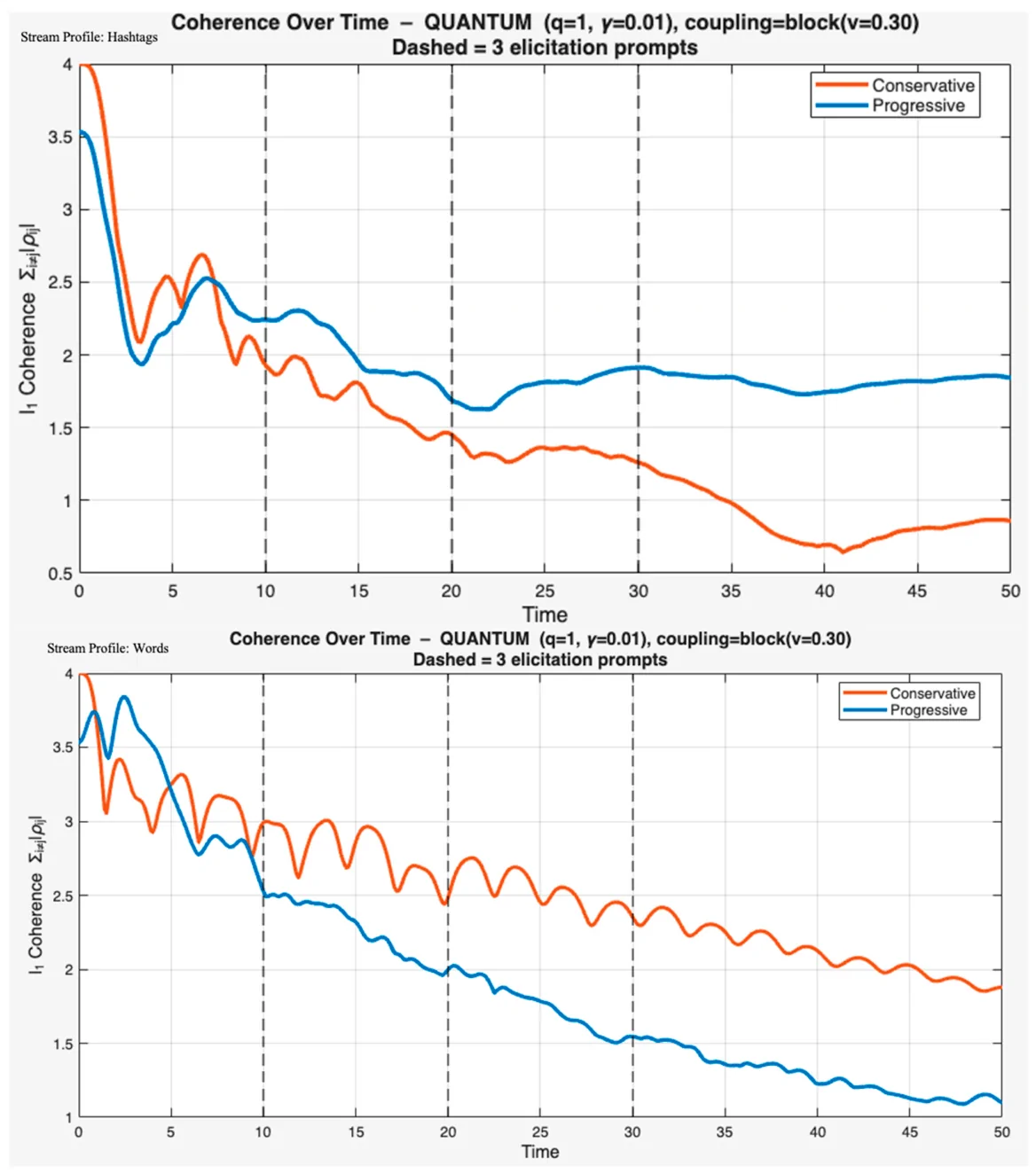
Coherence over time full quantum heavy defense (γ=0.01).

In this set of conditions, although there is no Markov component for the attack, there is a heavy defense component which is Markovian. As can be seen in Figure 8, Figure 9 and Figure 10, the defense mechanics prevents oscillation and pulls the final distribution towards the prior distributions for both conditions. Regardless of the information stream type, hashtag or word, the defense mechanism prevails, and the target audience preserves its perspective. As shown in Figure 8 and Figure 9, elicitation expedites the stabilization and minimizes the confusion.

**Figure 11:**
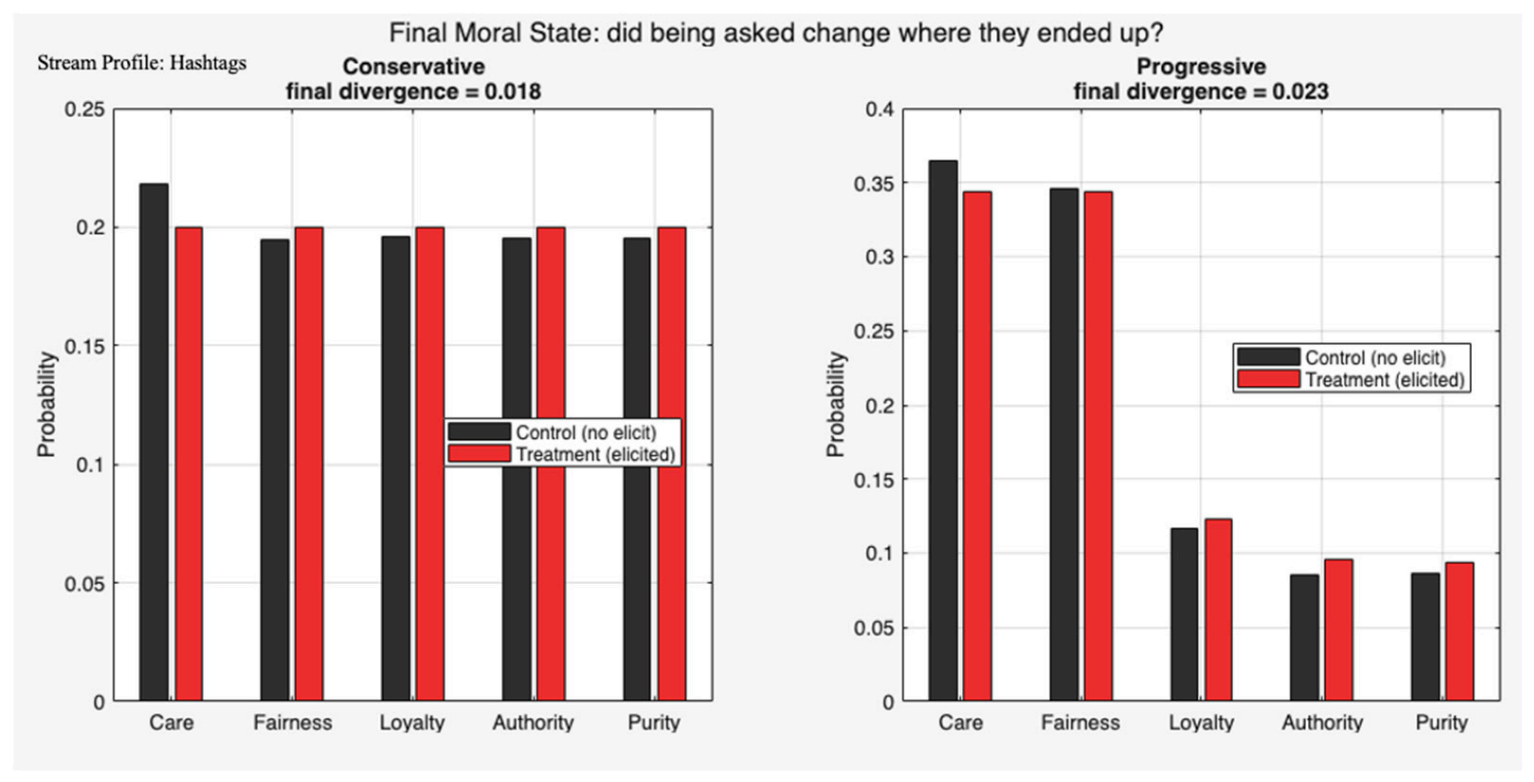

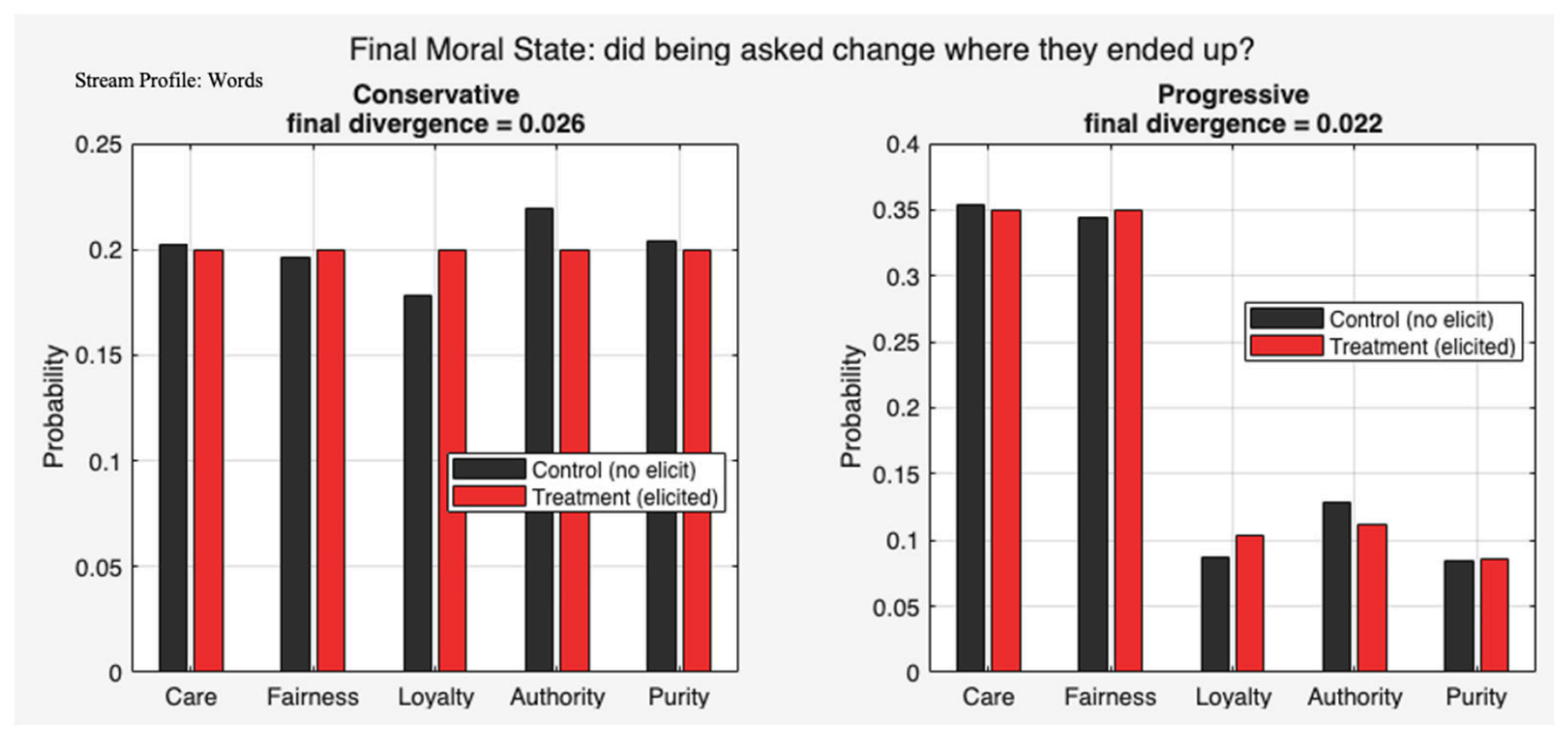
Final Probability Distribution for MFT pillars full quantum heavy defense (γ=0.01).

As shown in Figure 11, eliciting when prompted, improved the defense efforts, and final MFT probability distribution per pillar remained at the prior distribution levels.

### 4.3. Pure Classical Case #1 q=0, γ=1

**Figure 12:**
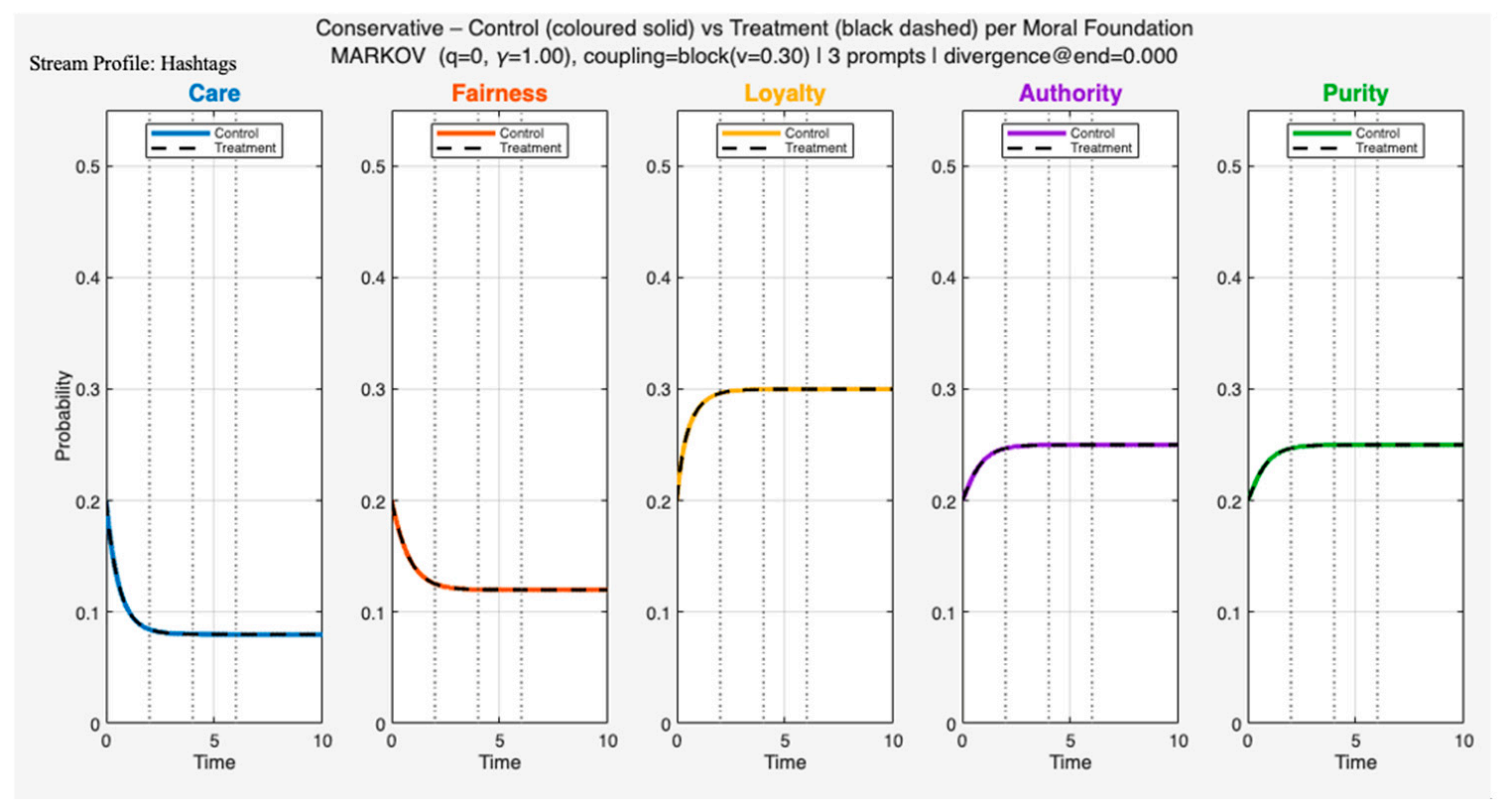

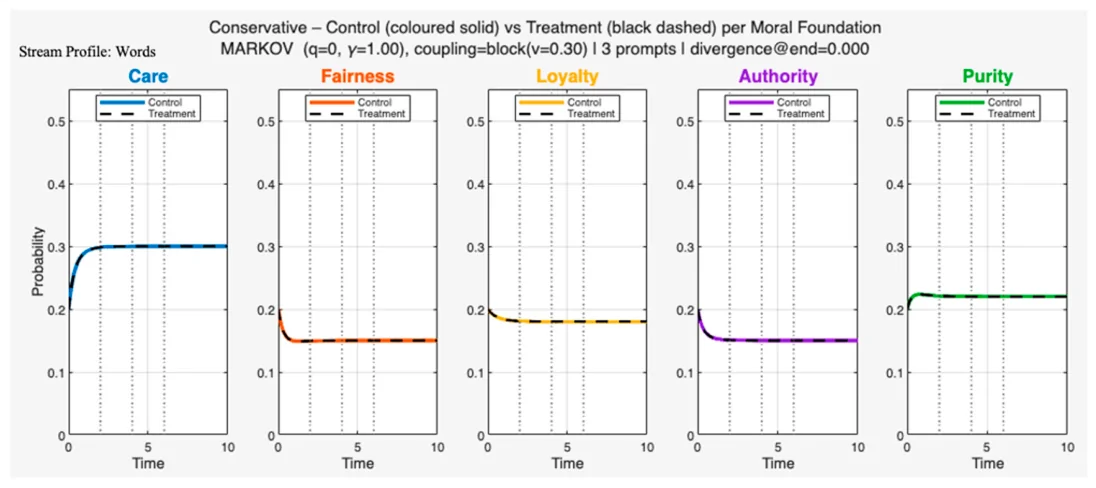
Conservative Probability distribution for Control vs. Treatment per MFT pillar.

**Figure 13:**
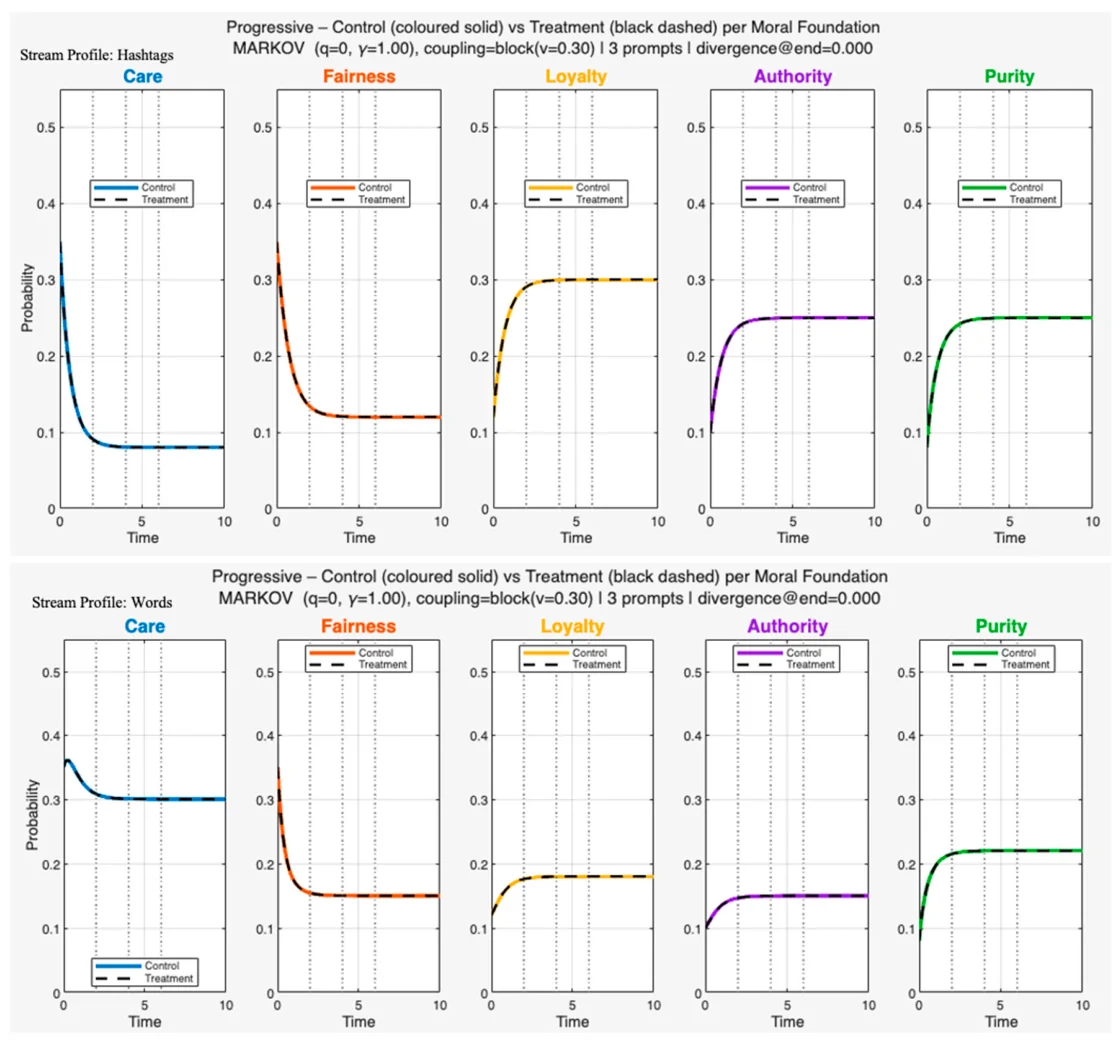
Progressive probability distribution for Control vs. Treatment per MFT pillar.

**Figure 14:**
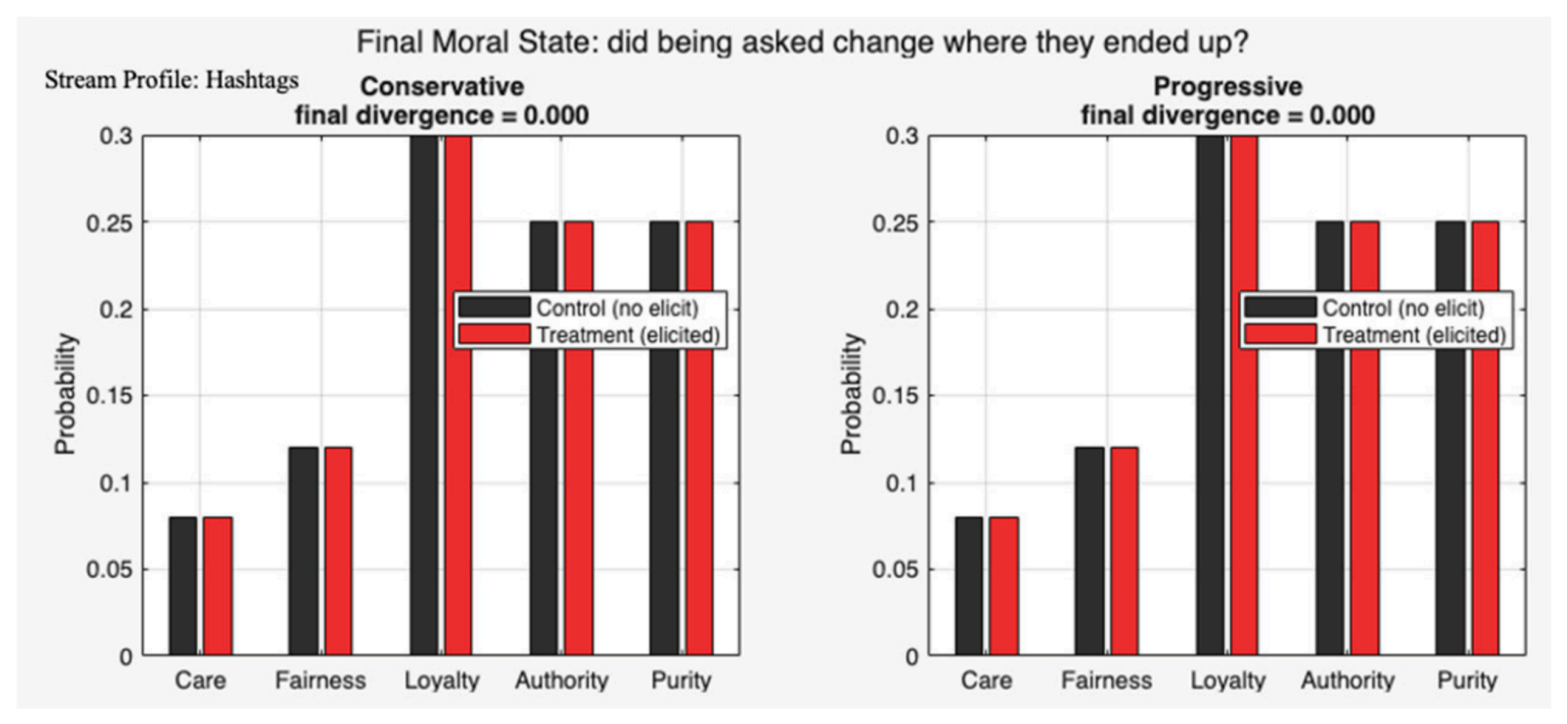

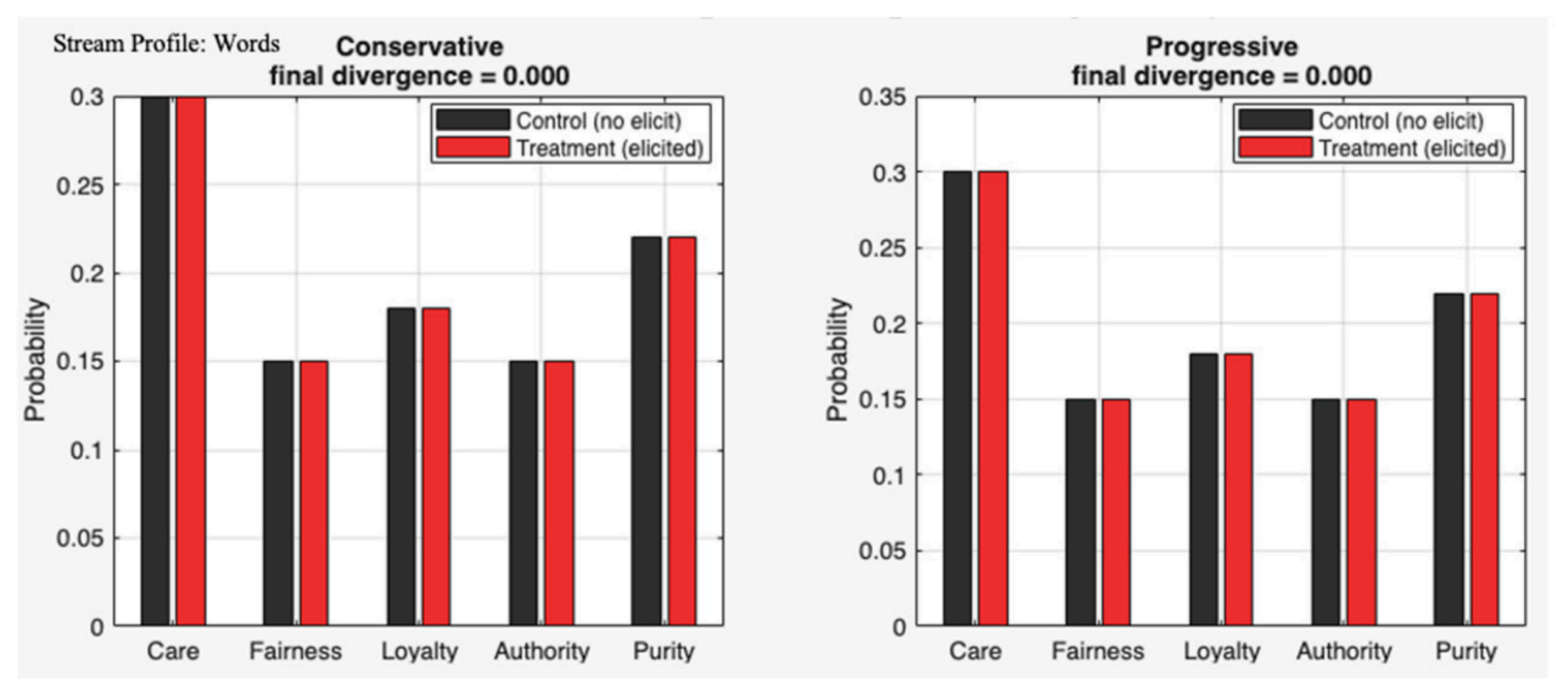
Final MFT state.

In this set of conditions, the developed model is tested for Markov dynamics of the attack component without having any defense resistance. The conservative group increased the probability distribution for the binding block MFT pillars shown in Figure 12; in both of the information stream cases, the target audience perspective adapts to the information stream distribution. For the progressive target audience, the same behavior is observed within the model simulation; for both of the information stream conditions, the MFT probability distribution adapts, as shown in Figure 13, to the stream’s distribution. Since there is no quantum contribution in the attack component, the treatment and control condition results are identical, as shown in Figure 14, and no oscillation has been seen in the model simulation. This shows the unique aspect of modeling human behavior with quantum dynamics.

### 4.4. Pure Classical Case #2 q=0, γ=0.01

**Figure 15:**
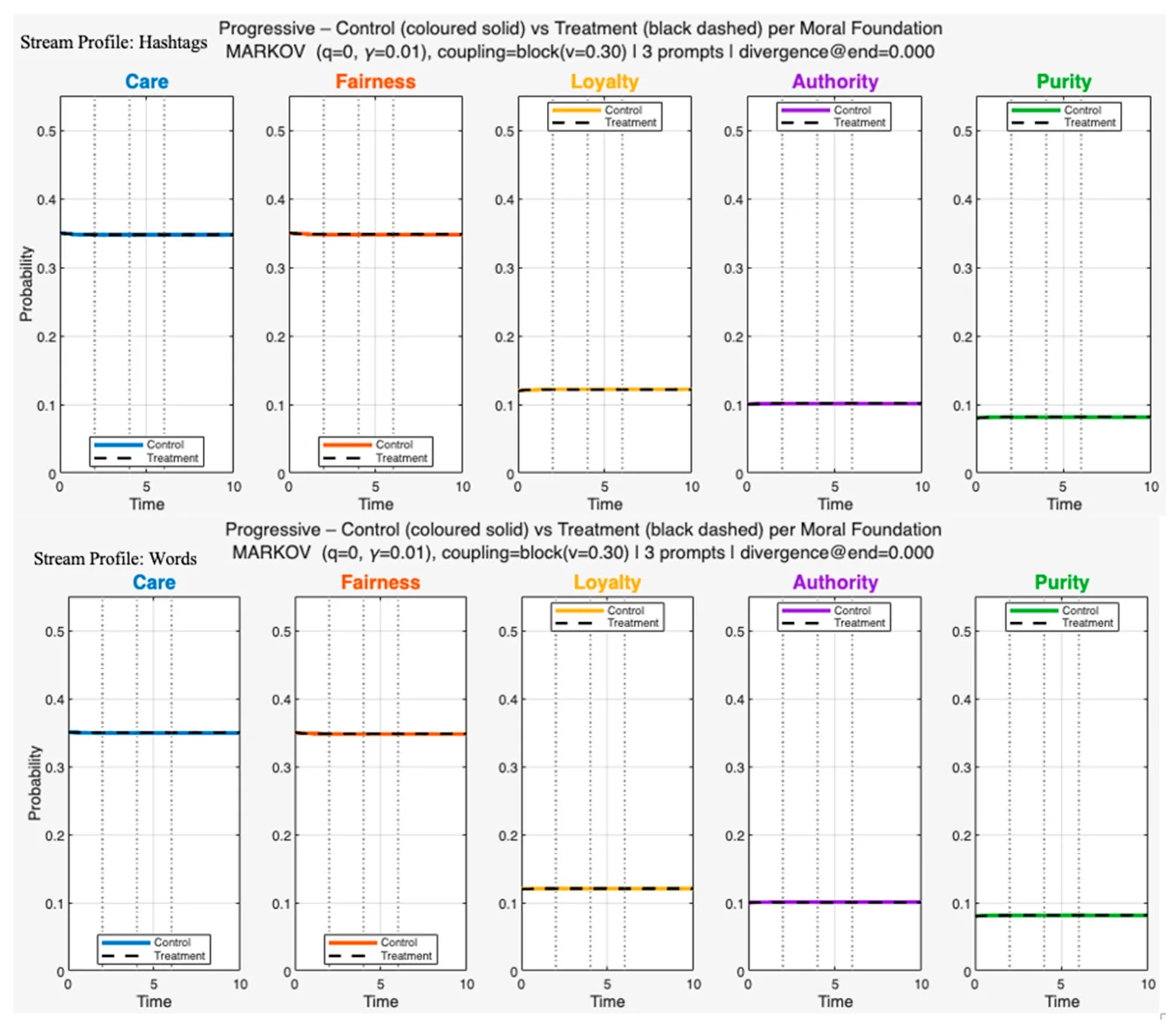
Progressive Probability distribution per Moral foundation full Markov q = 0 and high defense γ=0.01.

**Figure 16:**
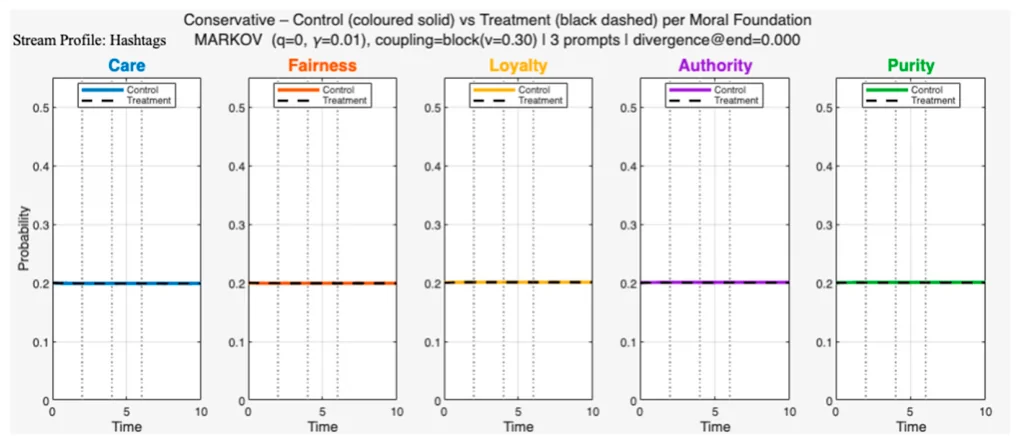

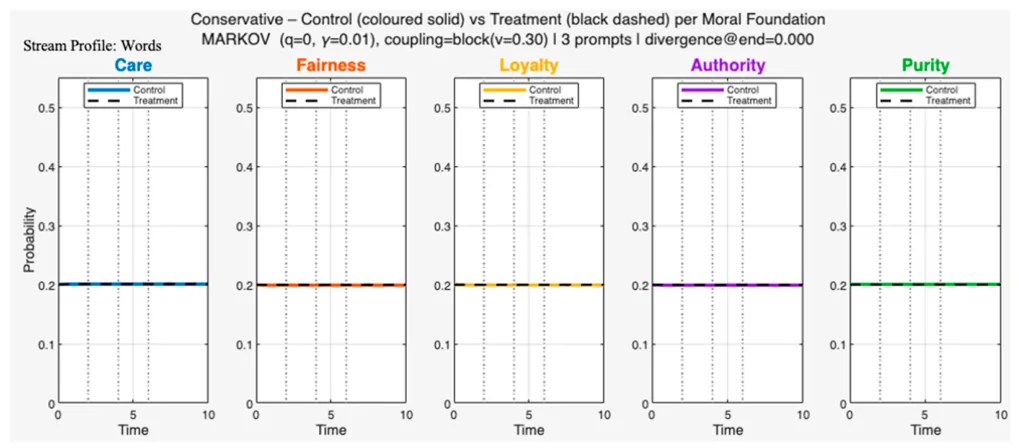
Conservative Probability distribution per Moral foundation full Markov q = 0 and high defense γ=0.01.

**Figure 17:**
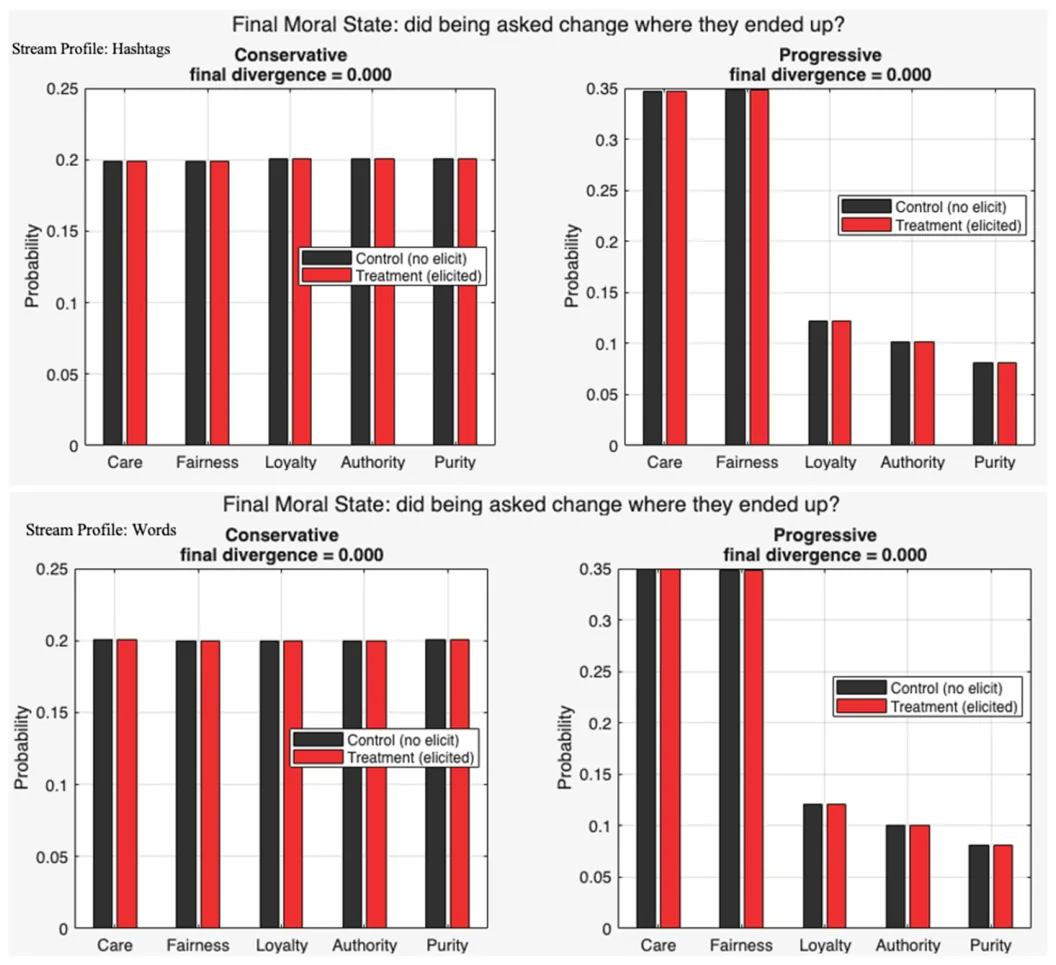
Final Moral State for full Markov q = 0 and γ=0.01.

In this set of conditions, what is tested is that the defense mechanism dominates the process and the only attack component is classical Markovian. As can be seen in Figure 15, Figure 16 and Figure 17, the system protects the prior distribution in all cases, and each target audience maintains its initial distribution regardless of the information stream. This is a checkpoint for the model’s defense mechanics, if that is consistent in terms of defending against a weak information exposure.

### 4.5. Full Quantum q=1 Balanced Attack and Defense γ=0.5

**Figure 18:**
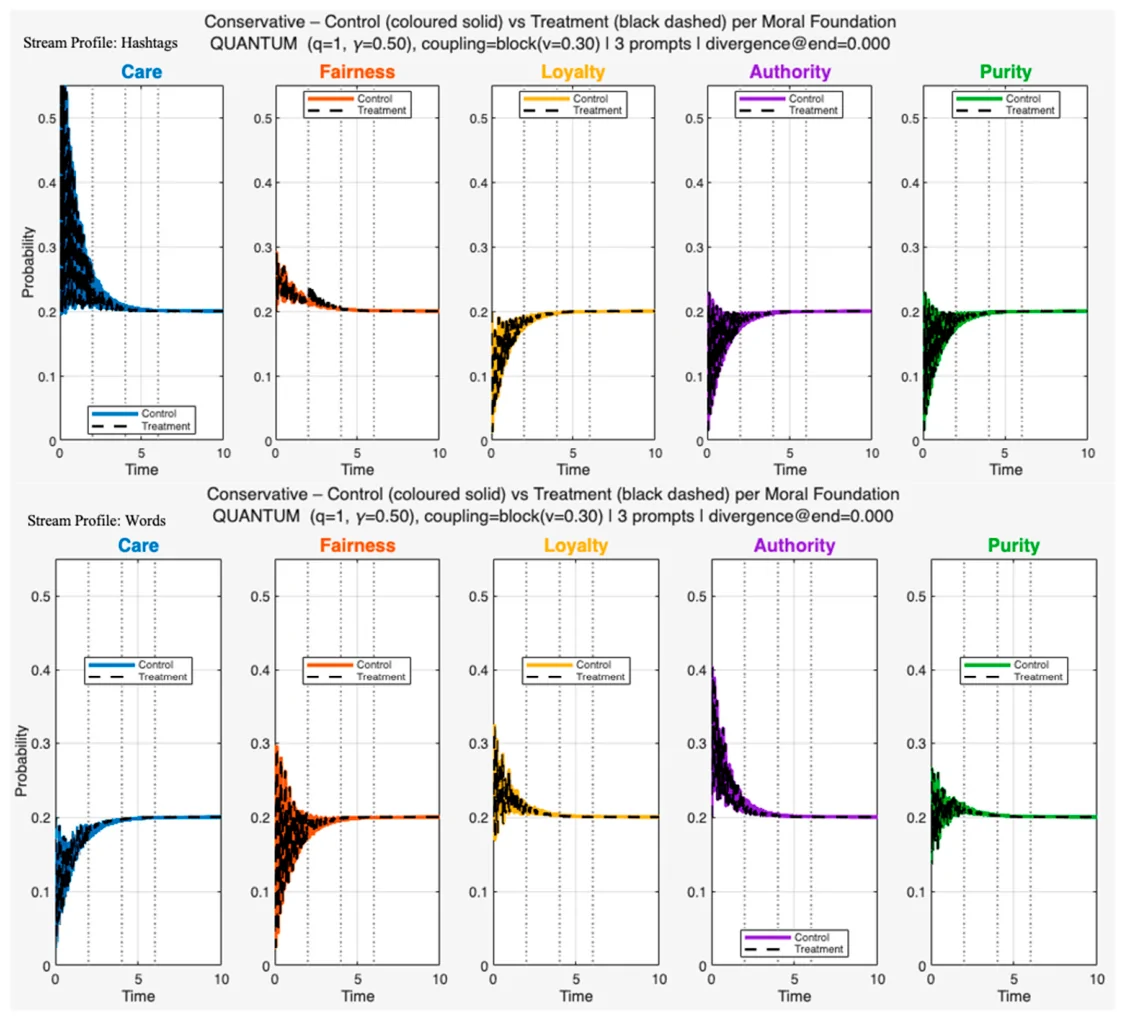
Conservative Probability distribution per Moral foundation full quantum q = 1 and high defense γ=0.5.

**Figure 19:**
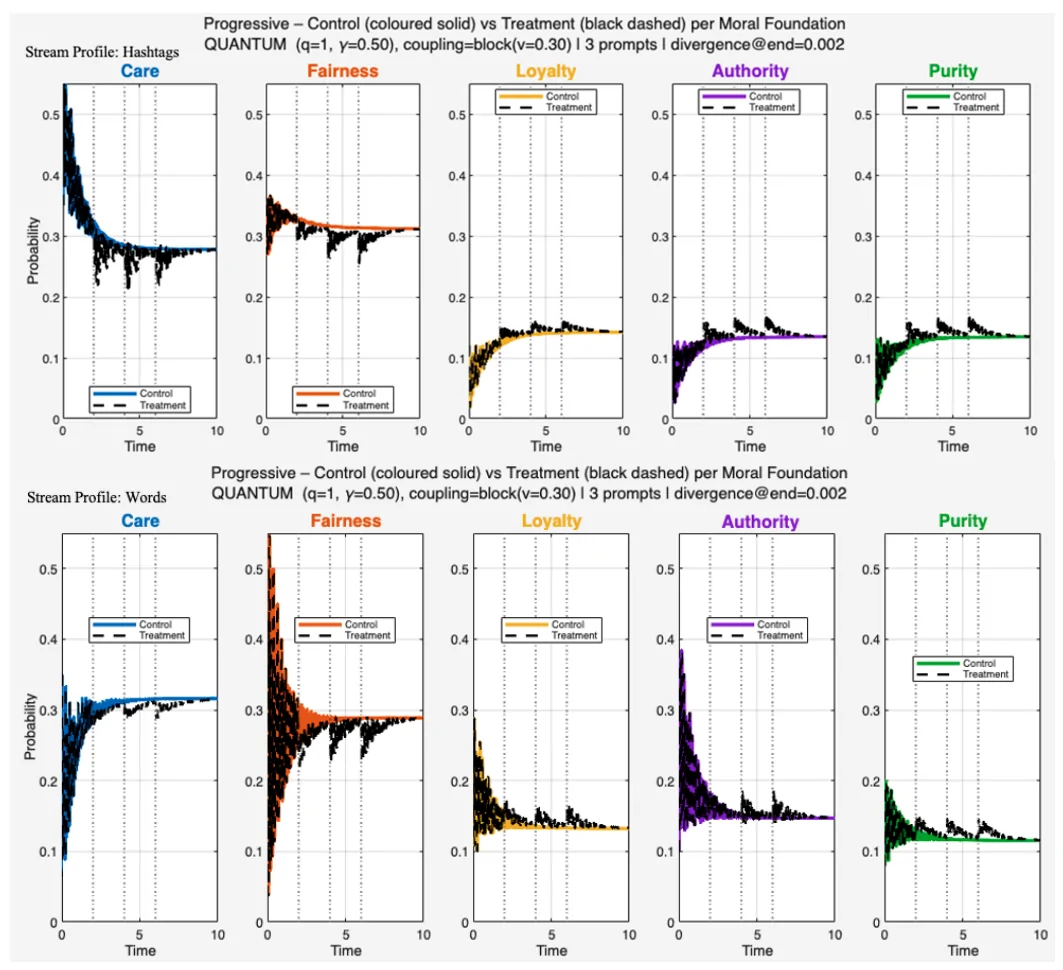
Progressive Probability distribution per Moral foundation full quantum q = 1 and high defense γ=0.5.

**Figure 20:**
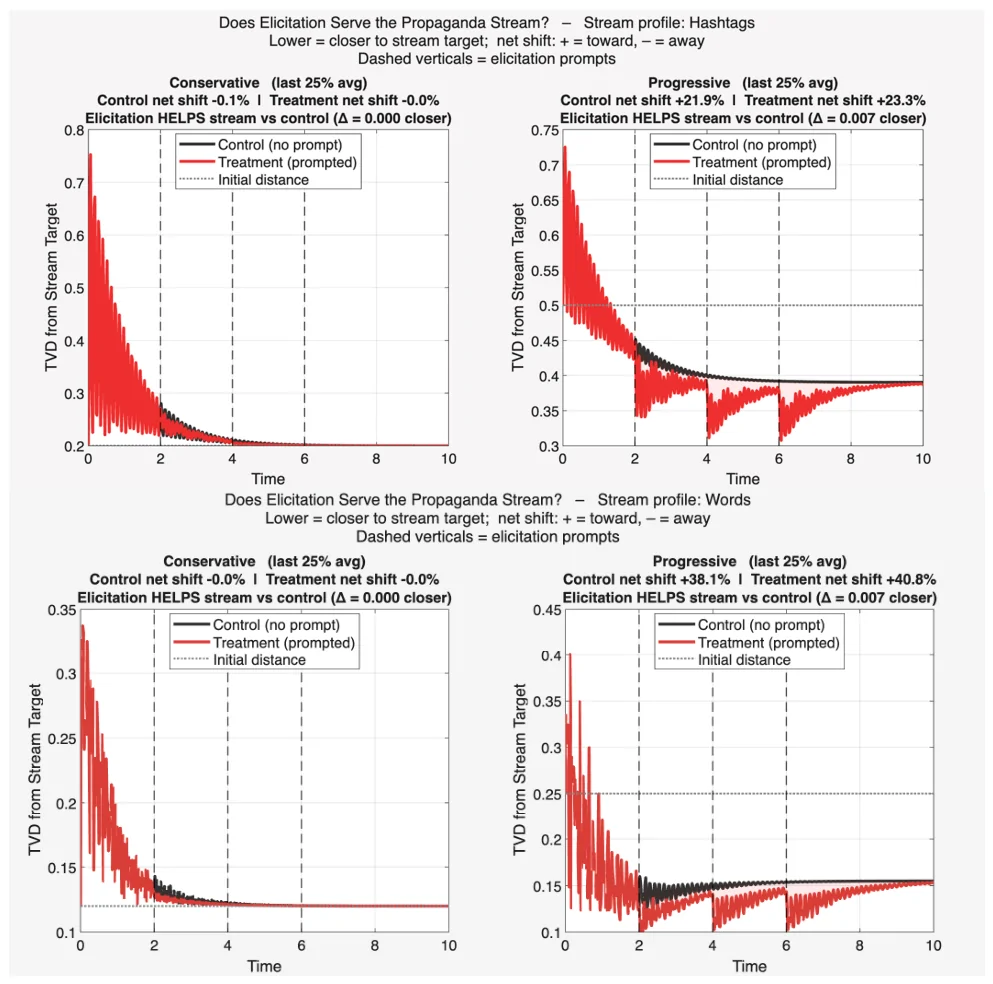
Total Variation Distance (TVD) between target audience initial distribution and propaganda stream q = 1 γ=0.5.

**Figure 21:**
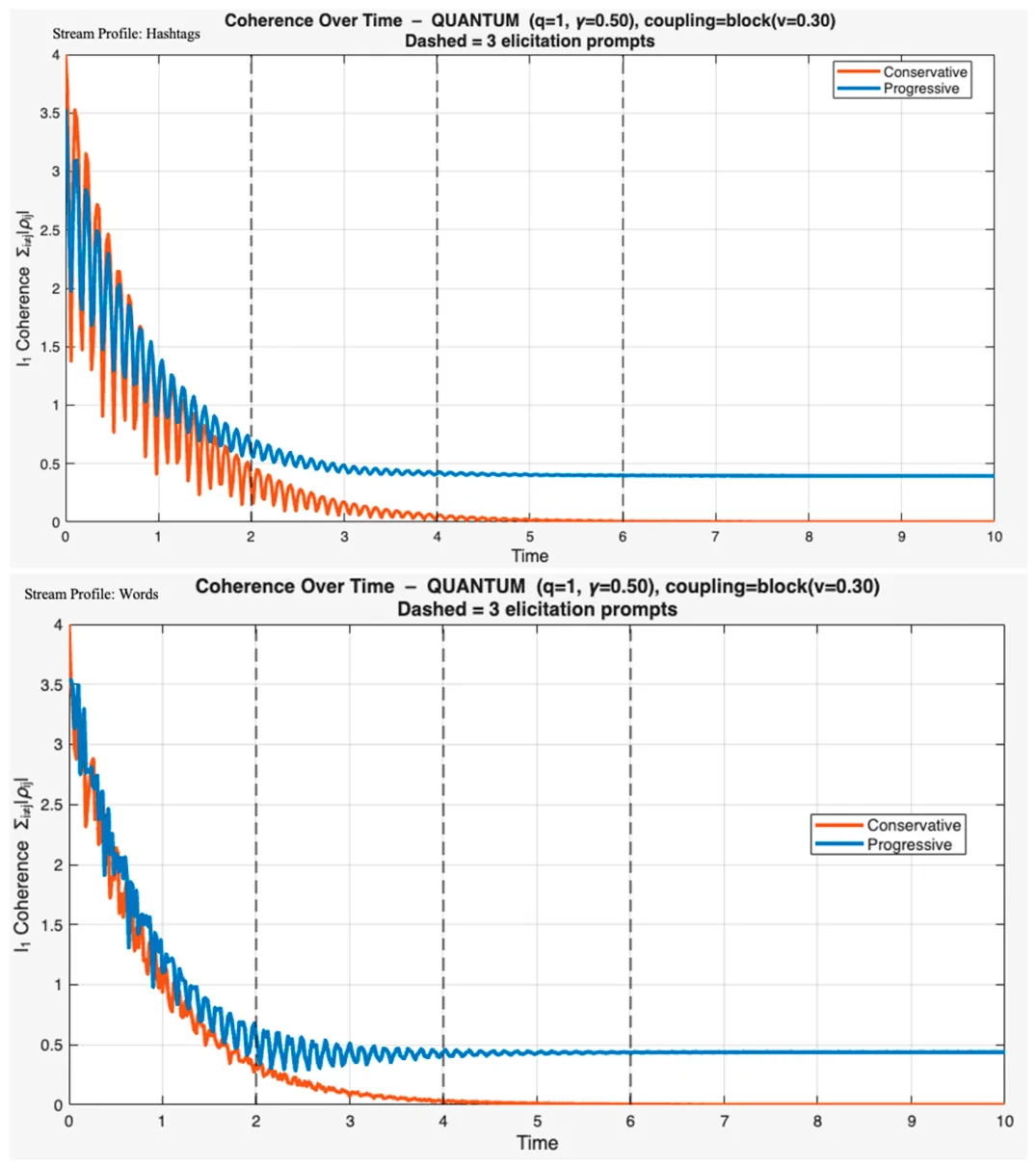
Final MFT pillar distribution for q = 1 and γ=0.5.

In this set of conditions, the attack component is dominated by the quantum dynamics, and the defense and attack components are equally weighted. Similar to the previous full quantum dynamic case, the non-selective elicitation serves the purpose of the propaganda. For example, as shown in Figure 18, elicitation helps to align the conservative prior to the stream, which is also conservative. However, as can be seen in Figure 19, the non-selective measurement, in the simulation model, changes the probability distribution in favor of the information stream. This simulation model indicates that the confusion element discussed in [54] is accentuated with the quantum model; when a target audience is confused, the subsequent prompted elicitation of a category in the form of non-selective measurement, within the model simulation, changes the outcome in favor of propaganda, which can also be seen in Figure 20 and Figure 21.

### 4.6. TVD Comparison for γ=0.5, q=[0.1−1.0]

So far in the analysis section, different sets of conditions were used to show how the model captures different behaviors. The model captures the critical characteristic of human behavior that is consistent with the earlier research in quantum open system modeling. In this section, 10 different conditions, shown in Table 4, were used to test the total variation distance outcome with the information stream set to Hashtags. As shown in Equation (10), the distribution generates more conversion with the progressive perspective, and elicitation helps to change a progressive perspective to a conservative perspective. However, since the information stream is not in a big conflict as it is with the conservative distribution, other full quantum cases, elicitation does not help the information stream.

**Figure 22:**
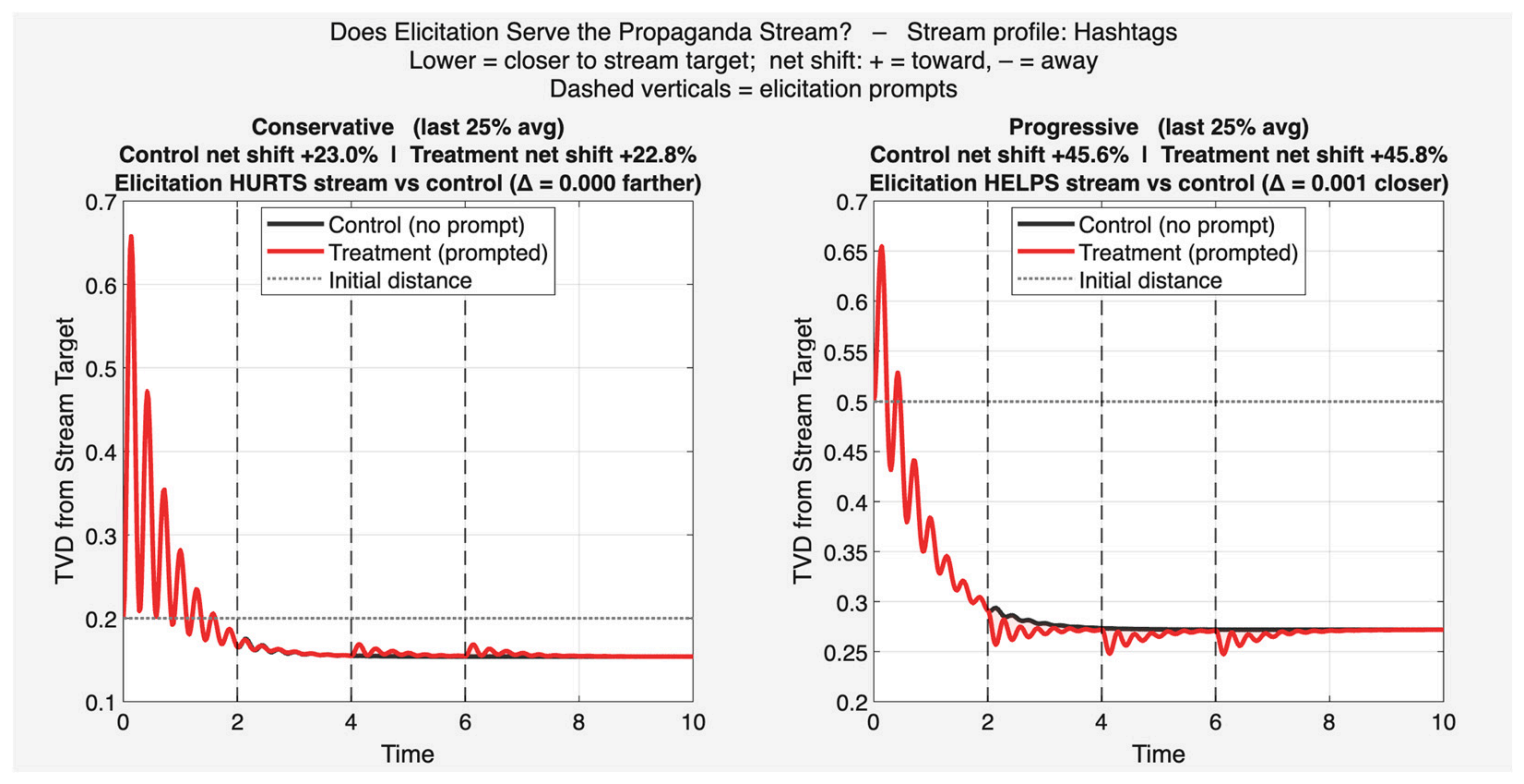
TVD γ=0.5 and q = 0.4.

Figure 22 shows a condition in which Markov attack dynamics are 20% more dominant than the quantum dynamics. As expected, relatively smaller oscillations for both treatment and control groups have been observed in the model simulation; however, due to the presence of quantum dynamics, elicitation in the simulation model, via non-selective measurement, pushes the progressive target audience towards the information stream distribution, and the target audience perspective becomes more aligned with the conservative perspective, all of which are within the simulation model.

**Figure 23:**
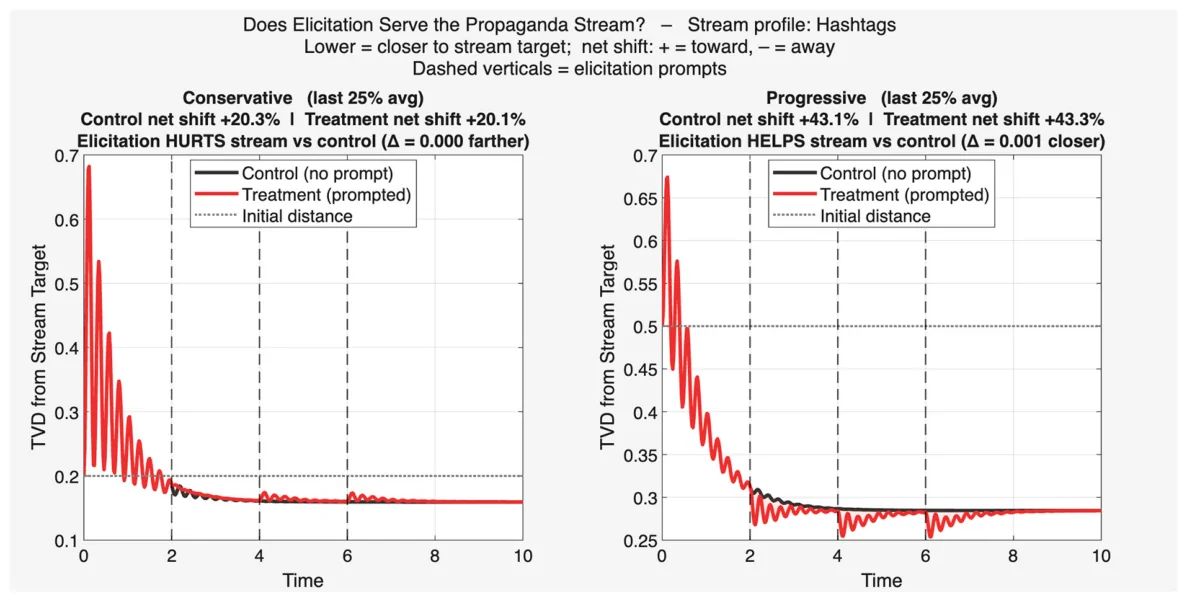
TVD γ=0.5 and q = 0.5.

Figure 23 shows a balanced attack condition, and the sensitivity of the progressive group to non-selective elicitation is still higher compared with the conservative group. This is expected because the information stream is already conservative. The key observation for the conservative group is that non-selective elicitation allows the target audience to preserve the probability distribution within the binding block.

**Figure 24:**
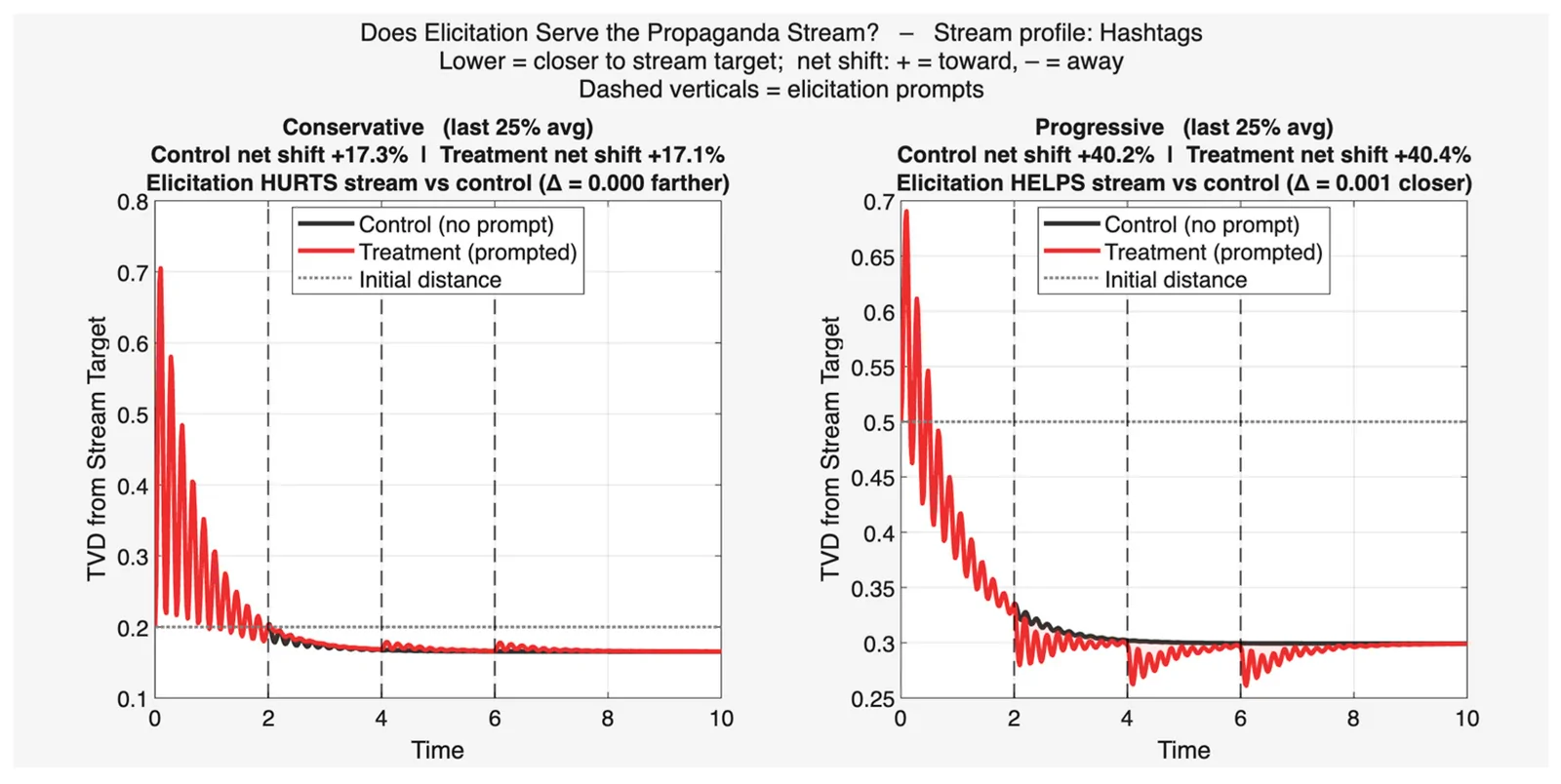
TVD γ=0.5 and q = 0.6.

Figure 24 shows a case in which quantum attack components are more dominant over the Markov attack component. Figure 24 demonstrates that in the case of having higher oscillation, the elicitation, in the simulated dynamics, helps the stream such that the progressive perspective becomes aligned with the conservative perspective.

The non-selective measurement, in this simulation study, gives a boost to the intended effect of the information stream in the case of the progressive target audience more than the conservative target audience. For the conservative audience, the partial quantum attack, q<1, is more comprehensive than the fully quantum one q=1, because the classical channel keeps pushing while the pure quantum (dominated by Hamiltonian dynamics only) attack freezes at the maximum mixed point. Maximum quantumness is not maximum propaganda effectiveness for a uniform-prior audience.

A propaganda operation using a highly coherent attack, which aims to generate/exploit ambiguity, can improve the intended outcome by prompting engagement more frequently and focusing earlier and altering prompting; each prompt collapses the ambiguity (off-diagonal elements become zero) before it can resolve back, pulling the audience toward the stream. High-frequency engagement/prompting can be a way to convert oscillatory destabilization into directional capture.

## 5. Conclusions

This paper demonstrated that MFT can be used in an interaction model to represent a cognitive system and information stimuli. The model delivered in this research can be used to analyze the information interaction activities in the information environment; the effects of information interaction can be simulated with this model. The main drawback of the common approaches is that there is no unified framework to represent the schema of the target audience and the schema of the information stream with the same basis for the system states. Using the five-pillar psychological system of the moral foundation theory to represent the system states in the interaction introduces a framework to analyze and study adaptation, intuition, and environmental influences in a systemic way in the context of disinformation propaganda. The unique aspects of the model are as follows. First, the model accounts for both the quantum and classical dynamics. Second, the information stimuli, attack mechanics, defense mechanics, and the cognitive system are represented on a single common basis, the five-dimensional moral foundation state space. The introduced model supports an adjustable attack vector for an information stream and defense vector for the cognitive system. The model is capable of representing confusion (via coherence that captures ontic type uncertainty) of a target audience that is exposed to an information stream. As discussed, creating confusion is one of the characteristics of propaganda in the information age. The level of confusion can be adjusted by the parameters of the model, such as q,γ, v, λ. It should be noted that the model of this work predicts mechanisms and dynamics (in the simulation model), and more importantly, these mechanisms and dynamics have not been validated or tested against measured audience responses, within the simulation results of the model, and the Twitter data does not include information to use for this purpose. Consequently, the model developed in this study is an inference tool, not a source of statistical evidence about experimentally observed events. This inference tool is to elucidate mechanisms/dynamics when such cognitive dynamics could arise. Empirical validation of the mechanisms and dynamics remains a task for future research, which is discussed in Section 6.

The mechanisms/dynamics identified in this paper can have implications outside the model and be summarized as follows. (1) The amplifying effect of non-selective elicitation implies that information-disseminating platforms’ features designed to prompt engagement can function as elicitation events as described in this model. (2) The word frequency analysis in Appendix A shows the most frequently used words in the Twitter data. However, from an information-processing and interaction perspective, the dynamics and mechanisms demonstrated in this model support a new perspective on the interaction model. This work argues that effective countering requires engagement at the level of generalizable framing, such as MFT, rather than vocabulary content. (3) A model of target-audience vulnerability certainly can be dual-use. For example, the same analysis that supports defense can inform targeting. These are stated as risks and mechanisms/dynamics, not as operational recommendations, because the simulation model predicts mechanism/dynamics and requires validation against measured audience responses. In the simulation model, recommendations for platform operators would require empirical evidence, which can be done with future research.

The mechanisms/dynamics introduced in this work identify potential safeguards that are quantifiable in the parameters of the model. For example, interface contact that prompts deliberation before engagement would mean requiring an article to be opened before resharing. This can be considered as a possible design approach to reduce the frequency of elicitation events, and the results of this paper predict their simulated effect; for example, less frequent elicitation substantially weakened capture by the information stream. Designs that introduce a delay before response can be related to increased relative weight on the defense channel, under which the cognitive state relaxes toward the audience’s prior between exposures. It should be noted that these are mechanism/dynamics derived hypotheses about intervention, not validated recommendations. This study, within the model, demonstrates that the predicted simulation effects follow from the model; their real-world behavior is an empirical question belonging to future empirical work, and their implementation involves trade-offs between safeguarding and expression that require normative analysis beyond a modeling study.

Without recognizing the psychological systems as a foundation to represent mental states of the individuals, the efforts to counter disinformation propaganda would not only fail but also carry the risk of inadvertently augmenting the intent of the disinformation propaganda. Since propaganda exploits the cognitive peripheral routes, an approach that is supported by MFT will ameliorate the design of the counter-disinformation operation with a comprehensive framework. Removing a node in a network or the content from the World Wide Web will not undo the impacts of the disinformation propaganda from the cognitive dimension. Moreover, ignoring this aspect means that individuals in free societies can become more vulnerable to threats in the information ecosystem.

## 6. Limitations and Future Research

This study is a theoretical model-development study. The developed model has not been tested with any data. The developed model builds on previous open system modeling techniques [48] and introduces a new method to monitor the complete positivity of the ρ density matrix for cognitive modeling. The presented model includes the information stream, the attack-defense balance, and the quantum-classical balancing elements over the evolution. However, this has not been tested with any human subject experimentation. Therefore, the limits of the claim are at the theoretical level. The target audiences studied are likewise idealized, such as the conservative and progressive profiles, which are ensemble-level characterizations drawn from published MFT survey research, not populations measured from the platform data. The two-profile (conservative vs. progressive) design of the model abstracts away in-group heterogeneity and from any moral contexts specific to different communities, including ethnic-minority contexts, and no conclusion attaches to any individual user. It should be noted that the uniform profile extracted from MFT literature lies at the center of the moral foundation spectrum, hence, the design of the information stream in the model spans the moderate-to-skewed range rather than opposing ends. The Twitter data serves to extract the moral foundation profile of a real information stream. The data set does not contain any behavioral or demographic information about exposed users, as a result, it characterizes the stimulus rather than validating the model’s predicted responses in the simulation, for example, in the case of elicitation. A stated bridging assumption underlies the stimulus representation that the relative frequency of moral framings in the stream translates into the relative strength of the bias exerted on the cognitive dimension, on the other hand, word frequency characterizes the content of the broadcast, not the content received by the information processing entity.

Appendix B establishes GKSL validity uniformly across the parameter conditions studied. In real-time information operations, the parameters vary in time; for example, stream strength changes with campaign dynamics, or the target audience’s defense strength varies as well. In such time-varying settings, validity is no longer guaranteed by a single theorem; in fact, it becomes a property of each evolution interval, and the Choi criterion (see Appendix C) introduced into the model is the instrument that extends the framework to that regime.

This study makes no empirical claims about audience responses; however, the introduced Choi criterion, together with the introduced model, allows (as part of future empirical work) an empirical testing of a question, that is, “is target audience cognition under a continuous information stream Markovian, or is it history dependent, exhibiting phenomena such as inoculation?” This question can be tested with data because, in the case where CP violations are present, they become an indicator of memory effects [72,73]. Two extensions can be derived from the results of this study. (1) The vulnerability asymmetry reported here should be tested for symmetry. For example, exposing the conservative profile to a Care/Fairness-dominant stream would determine whether the observed sensitivity in the model simulation is a property of a mismatch between audience and stream or solely a property of an audience profile. (2) The metrics reported in this paper are total variation distance (TDV) and net shift. These metrics quantify only an audience’s shift toward the information stream distribution. These two metrics fail to distinguish other forms of displacement. For example, a state may move toward moral profiles that belong to neither the audience’s prior nor the profile of the information stream. Therefore, developing displacement measures that can separate alignment with the stream from such off-target displacement, and relating them to coherence-based measures of ambiguity/confusion, is the next step for this study.

## Figures and Tables

**Table 1:** Moral foundation pillars.

Pillar	Description
Harm/Care	Basic concerns for the suffering of others, including virtues of caring and compassion.
Fairness/Cheating	Concerns about unfair treatment, inequality, and more abstract notion of justice.
Ingroup/Loyalty	Concerns related to obligations of group membership, such as loyalty, self-sacrifice and vigilance against betrayal.
Authority/Subversion	Concerns related to social order and the obligations of hierarchical relationships, such as obedience, respect, and proper role fulfillment.
Purity/Degradation	Concerns about physical and spiritual contagion, including virtues of chastity, wholesomeness and control of desires.

**Table 2:** Seed words were used to generate prototypicality estimates in [25].

Valence	Foundation				
	Care	Fairness	Loyalty	Authority	Sanctity
Virtue	kindness compassion nurture empathy	fairness equality justice rights	loyal team player patriot fidelity	authority obey respect tradition	purity sanctity sacred wholesome
Vice	suffer cruel hurt harm	cheat fraud unfair injustice	betray treason disloyal traitor	subversion disobey disrespect chaos	impurity depravity degradation unnatural

**Table 3:** Model parameter for the simulation analysis. Coupling and $V_{0}$ values are chosen to be consistent with [48].

γ	q	$\lambda_{attack}$	$\lambda_{defense}$	Coupling	$V_{0}$	v
1	1	5	5	35	500	0.3
0.01	1	5	5	35	500	0.3
1	0	5	5	35	500	0.3
0.01	0	5	5	35	500	0.3
1	0.5	5	5	35	500	0.3

**Table 4:** q vs. Shift for control and treatment conditions.

	Net Shift (Information Stream: Hashtag)			
	Control Condition		Treatment Condition	
q	Conservative	Progressive	Conservative	Progressive
0.1	33.7%	50.9%	33.6%	51.0%
0.2	28.8%	49.6%	28.7%	49.7%
0.3	25.7%	47.8%	25.6%	47.9%
0.4	23.0%	45.6%	22.8%	45.8%
0.5	20.3%	43.1%	20.1%	43.3%
0.6	17.3%	40.2%	17.1%	40.4%
0.7	13.9%	36.7%	13.7%	37.1%
0.8	10.0%	32.7%	9.9%	33.3%
0.9	5.4%	27.8%	5.3%	28.8%
1	−0.1%	21.9%	0.0%	23.3%

**Figure A1:**
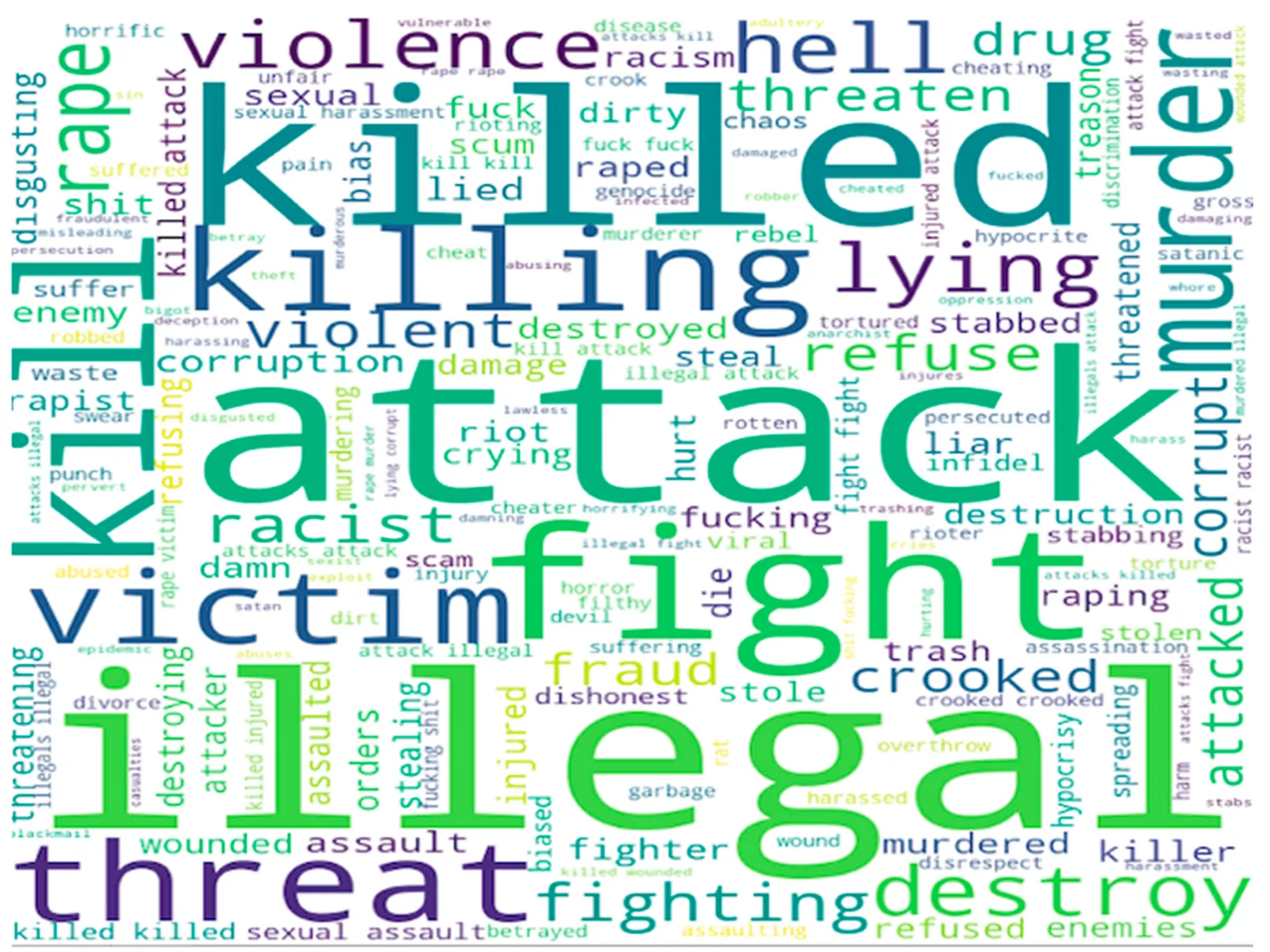
Moral foundation dictionary word cloud for the harm foundation.

**Figure A2:**
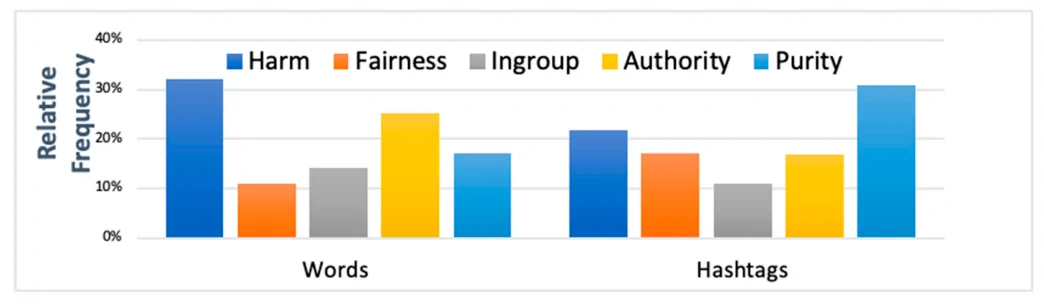
Moral foundation dictionary analysis of the Russian propaganda.

**Figure A3:**
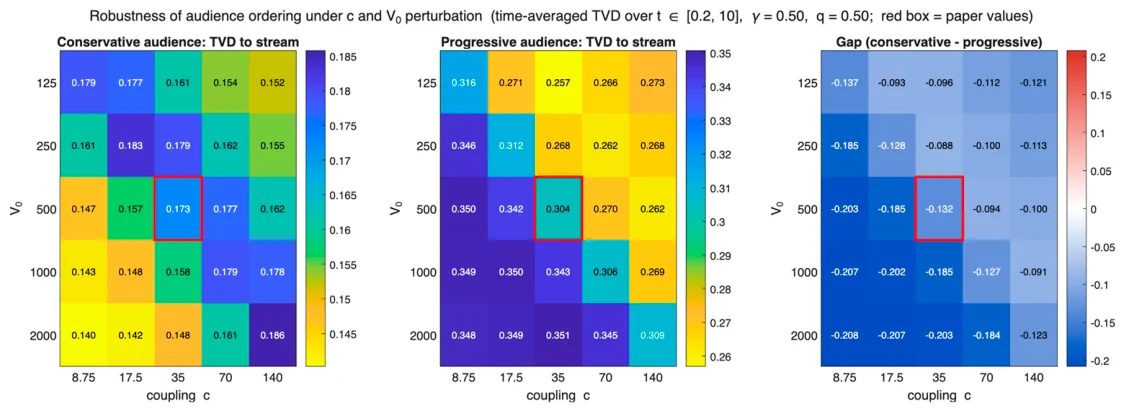
The averaged distance for both the conservative (**left**) and progressive (**center**) audiences and the difference between conservative and progressive (**right**).

## Data Availability

The data presented in this study are openly available at https://blog.x.com/en_us/topics/company/2018/enabling-further-research-of-information-operations-on-twitter (Accessed/downloaded on 30 June 2023) and https://www.kaggle.com/datasets/paultimothymooney/twitter-election-data-archives (Accessed/downloaded on 30 June 2023).

## Appendix A. Analysis of Twitter Data

On 31 January 2018, Twitter (now X) released a comprehensive archive of tweets and media associated with state-sponsored information operations. The importance of this data set is that it includes entities that knowingly attempted to manipulate and distort public conversation regarding the 2016 U.S. presidential elections as part of an information operation. This data set is categorized as an information operation by Twitter; therefore, analysis of this data set can be conducted without a study to determine whether this data set is part of an information operation or not.

### Appendix A.1. Analysis of Information Operation Data

First, the algorithm that was developed as part of this work was tested with the MFT dictionary by analyzing the morality of random tweets related to UNICEF and Sky-Sport-Boxing. The results indicate that 74.41% of the @UNICEF-related tweets are virtue-oriented, and 88.95% of the @skysportboxing-associated tweets are vice-oriented; thus, both the dictionary and algorithm are sensitive enough to capture the morality of tweets.

The largest data set in the January 2018 release was selected. This data set includes IRA-associated accounts, tweets, and media. The total number of tweets in this data set is 920.

1. The analysis is conducted with the English version of the MFT dictionary, and after removing the tweets that contain only hashtags, the total number of English tweets becomes 592,360.

After extracting the repeated words and hashtags in Table A1 and Figure A1, the repeated words are compared with the words [13] that were commonly used in the 2016 presidential election campaigns. Although this dataset was extracted from an information operation that intended to manipulate the 2016 U.S. presidential elections, the repeated words in Table A1 and Figure A1 have sparse relation to both of the campaigns. For example, from the word count, only “hillary” and “obama” could be related to one of the campaigns, and from the hashtag list, “maga” and “americafirst” hashtags can be related to a campaign. A target audience analysis that is based on word frequency cannot be used to build an interaction model for two reasons. First, the word frequency is unique to an information stream and cannot be used to represent an audience. Second, using words limits the model to the words, not for a generalizable target audience.

**Table A1:** Number of repeated words and hashtags in English Tweets.

Words (Number of Repetition > 20,000)		Hashtags (Number of Repetition > 10,000)	
islam	46,818	maga	40,068
today	31,669	releasethememo	37,699
obama	31,327	islamistheproblem	21,666
lesson	29,531	bansharialaw	17,332
hillary	25,822	Stopimportingislam	15,285
say	25,342	banislam	12,468
muslim	23,333	americafirst	11,713
via	23,029	islam	10,005

A hashtag frequency analysis was conducted because, contrary to Twitter’s recommendation of using two hashtags in a tweet to maximize visibility, there are thousands of tweets with 3, 4, 5, and 6 hashtags.

### Appendix A.2. MFT Dictionary Analysis

IRA-associated tweets were further analyzed by using the MFT English dictionary [25]. After cleaning up the stop words, the remaining words were checked with the MFT dictionary clusters [25], and the associated seed words are listed in Table 2. The total number of tweets that include at least one moral dictionary word is 215,691, and the relative frequency distribution of the tweets with respect to moral pillars is shown in Figure A1. The co-occurrence analysis allowed one to check the number of tweets that include at least one of the most repeated (>10,000) words and the moral dictionary words; 97,990 tweets satisfy this condition. The context in this information stream can be gleaned by the individuals who have a similar moral matrix Figure A2.

The morality of the hashtags in this information operation can provide additional insight into the target audience analysis. Since hashtags are used to increase the visibility of a tweet [74], the frequency analysis of the moral dictionary can delineate the moral matrix of the target audience. In reference to the MFT clusters in Figure A2, the morality of hashtags in Figure A2 indicates a target audience towards the center of the moral spectrum.

By using the moral foundation analysis of the words and hashtags, one can argue that this specific information operation was targeting the moderate voters. However, the contribution of this approach, from the modeling perspective, is that both the schema of the information stream and the schema of the target audience can be expressed with the same framework; hence, they can be accounted for in systemic threat vector analysis that captures the interaction between the target audience and the information stream.

## Appendix B. Validity of the Composite Generator $L_{\mathrm{total}}$

The super-operator $L_{total}$ defined in Equation (13) is a (mathematically) valid generator of quantum dynamics for every parameter pair $\left(\gamma ,q\right)\in {\left[0,1\right]}^{2}$. It is shown that $L_{total}$ is in the Gorini–Kossakowski–Sudarshan–Lindblad (GKSL) standard form, from which it follows that the induced evolution map is completely positive and trace-preserving (CPTP), and therefore, for all conditions, the introduced super-operator maps density matrices to density matrices. Although this CP validity is based on proof and no numerical analysis is required, the CP simulation check is further explored, and additional proof of Choi matrix criterion application is discussed in Appendix C.

### Appendix B.1. Setting and Notation

The cognitive state space is the Hilbert space $H=C^{5}$ spanned by the orthonormal moral foundation basis { |i⟩:i=1,…,5 }, corresponding to the pillars Care, Fairness, Loyalty, Authority, and Purity. Matrix units are written $E_{ij}=\left|i\rangle \langle j\right|$, where:

$$E_{ij}^{†}=E_{ji},\;\;E_{ij}^{†}E_{ij}=E_{jj}.$$

The state of the target audience is represented with a density matrix ρ, which is Hermitian, positive semidefinite, unit trace, whose diagonal entries $p_{i}=\rho_{ii}$ are the foundation probabilities; the off-diagonal entries $\rho_{ij}$ in which i≠j, are the terms that represent coherences that captures the oscillatory behavior.

For each classical channel μ∈{attack,defense}, the Kolmogorov generator $K_{\mu }$ is constructed from a target probability vector $\pi^{\mu }$ (the stream profile s for the attack channel, the audience prior $p_{0}$ for the defense channel), a rate constant $\lambda_{\mu }>0$, and the symmetric block-coupling matrix C is used:

$${\left(K_{\mu }\right)}_{ij}=k_{ij}^{\mu }=\lambda_{\mu }\;C_{ij}\;\pi_{i}^{\mu }\;\;\left(i\neq j\right),\;\;{\left(K_{\mu }\right)}_{jj}=-\sum_{i\neq j}k_{ij}^{\mu }.$$

Since $\lambda_{\mu }>0$, $C_{ij}\in \{v,1\}$ with v≥0, and $\pi_{i}^{\mu }\geq 0$, every off-diagonal rate satisfies $k_{ij}^{\mu }\geq 0$. Moreover, since the diagonal entries are the total outflow rates, so each column of $K_{\mu }$ sums to zero.

The dissipator associated with channel μ uses the off-diagonal matrix units as jump operators:

$$D_{\mu }\left(\rho \right)=\sum_{i\neq j}k_{ij}^{\mu }\left(E_{ij}\;\rho \;E_{ij}^{†}-1/2\left\{E_{ij}^{†}E_{ij},\;\rho \right\}\right)=\sum_{i\neq j}k_{ij}^{\mu }\left(E_{ij}\;\rho \;E_{ji}-1/2\left\{E_{jj},\;\rho \right\}\right),$$

where {A,B}=AB+BA is the anticommutator. The sum runs over i≠j only and the diagonal entries of $K_{\mu }$ do not appear as jump rates. Appendix B.6 of Appendix B shows that this restriction is necessary to build a cognitive model.

Consequently, the composite generator of Equation (13) is as follows:

$$L_{total}\left(\rho \right)=\gamma \left[\;q\;\left(-i\;\left[H,\rho \right]\right)+\left(1-q\right)\;D_{attack}\left(\rho \right)\right]+\left(1-\gamma \right)\;D_{defense}\left(\rho \right),$$

where H is the attack Hamiltonian of Equation (5) and $\gamma ,q\in \left[0,1\right]$ in which γ is the attack-defense weight and q is quantum-classical weight parameters.

### Appendix B.2. Main Theorem

**Theorem 1. GKSL validity.** 
*For all* $\gamma ,q\in \left[0,1\right]$ *and Hermitian* H*, the generator* $L_{total}$ *is of GKSL standard form. Consequently, the family* $\Phi_{t}=e^{t\;L_{total}}$*,* t≥0*, is a quantum dynamical semigroup. Each* $\Phi_{t}$ *is positive and trace-preserving, and* $\rho \left(t\right)=\Phi_{t}\left(\rho_{0}\right)$ *is a valid density matrix for every initial density matrix* $\rho_{0}$ *and for* t≥0.

**Proof.** By distributing the non-negative scalar pre-factors and merging the two dissipators, which share the same set of jump operators $\{E_{ij}{\}}_{i\neq j}$, one can get the following:

$$L_{total}\left(\rho \right)=-i\;\left[\tilde{H},\rho \right]+\sum_{i\neq j}r_{ij}\left(E_{ij}\;\rho \;E_{ij}^{†}-1/2\left\{E_{ij}^{†}E_{ij},\;\rho \right\}\right),$$

where an effective Hamiltonian and the effective rates are as follows:

$$\tilde{H}=\gamma \;q\;H,\;\;r_{ij}=\gamma \;\left(1-q\right)\;k_{ij}^{attack}+\left(1-\gamma \right)\;k_{ij}^{defense}.$$

where $\tilde{H}$ is Hermitian, which means H is a real symmetric matrix by construction. Equation (5) is a non-negative combination of the symmetric coupling matrix C and a real diagonal stream potential, and γq≥0 is a real scalar. Moreover, every effective rate is non-negative, meaning that $\gamma \left(1-q\right)\geq 0$ and 1−γ≥0 for $\left(\gamma ,q\right)\in {\left[0,1\right]}^{2}$, and $k_{ij}^{\mu }\geq 0$ as established in Appendix B.1, hence $r_{ij}\geq 0$ for all i≠j. Consequently, the merged expression is the diagonal GKSL standard form that is a Hermitian Hamiltonian part plus a dissipative part with traceless jump operators $E_{ij}$ and non-negative rates $r_{ij}$. The coefficient matrix, which is the Kossakowski matrix, of $L_{total}$ in the operator basis $\{E_{ij}{\}}_{i\neq j}$ is the diagonal matrix $diag\left(r_{ij}\right)≽0$, which is positive semidefinite. By the GKSL theorem, a bounded superoperator generates a CPTP semigroup *if and only if* it admits this form with a positive-semidefinite Kossakowski matrix; the semigroup $\Phi_{t}=e^{tL_{total}}$ is completely positive and trace-preserving for all t≥0. □

**Note 1:** This proof does not require the attack and defense channels to share a stationary distribution. The channels relax toward different targets, s and $p_{0}$; this only determines the location of the asymptotic balance point, not whether the dynamics is physical or not.

**Note 2:** The proof holds simultaneously for every point of the parameter square $\left(\gamma ,q\right)\in {\left[0,1\right]}^{2}$. All parameter sweeps while testing the simulation reported in the analysis take place entirely within the set of physically valid dynamics.

**Note 3:** The merged expression shows that the model is not merely a sum of individually valid pieces; rather, it is a single GKSL generator whose effective Hamiltonian $\tilde{H}=\gamma qH$ and effective rate matrix $r_{ij}$ interpolate between the attack and defense channels. The parameters γ, q, hence, act inside one generator rather than switching between separate dynamical laws.

### Appendix B.3. Trace Preservation

**Lemma 1.** 
$TrL_{total}\left(\rho \right)=0$ *for every* ρ*, as a result,* $Tr\rho \left(t\right)=1$ *for all* t≥0.

**Proof.** Because of the cyclicity, the commutator term traces to zero, $Tr\left[\tilde{H},\rho \right]=Tr\left(\tilde{H}\rho \right)-Tr\left(\rho \tilde{H}\right)=0$. For each dissipative term, cyclicity gives rise to:

$$Tr\left(E_{ij}\;\rho \;E_{ji}\right)=Tr\left(E_{ji}E_{ij}\;\rho \right)=Tr\left(E_{jj}\;\rho \right),$$

while the anticommutator contributes $1/2Tr\left(E_{jj}\rho \right)+1/2Tr\left(\rho E_{jj}\right)=Tr\left(E_{jj}\rho \right)$. The two contributions cancel term by term, so the dissipative part is traceless in this construct. □

### Appendix B.4. Embedding of the Classical Dynamics in the Populations

**Lemma 2.** 
*The populations* $p_{i}\left(t\right)=\rho_{ii}\left(t\right)$ *evolve under the dissipative part of* $L_{total}$ *according to the classical Kolmogorov equation*

$$\dot{p}\;{|}_{diss}=\tilde{K}\;p,\;\;\tilde{K}=\gamma \;\left(1-q\right)\;K_{attack}+\left(1-\gamma \right)\;K_{defense},$$

*including the correct diagonal, outflow, entries of* $\tilde{K}$*, although only the off-diagonal rates enter the dissipator as jump rates*.

**Proof.** If the matrix elements of the dissipative part on the diagonal are evaluated for the sandwich term,

$$\langle i\left|\;E_{kj}\;\rho \;E_{jk}\;\right|i\rangle =\delta_{ik}\;\rho_{jj},$$

so the jump $E_{ij}$ with rate $r_{ij}$ pushes population from pillar j into pillar i at rate $r_{ij}\;p_{j}$. For the anticommutator term,

$$\langle i\left|\;\{E_{jj},\rho \}\;\right|i\rangle =2\;\delta_{ij}\;\rho_{ii},$$

so the same jump removes population from pillar j at rate $r_{ij}\;p_{j}$. Summing over all jumps,

$${\dot{p}}_{i}\;{|}_{diss}=\sum_{j\neq i}r_{ij}\;p_{j}-\left(\sum_{k\neq i}r_{ki}\right)p_{i}=(\tilde{K}\;p{)}_{i},$$

which is precisely the classical master equation with generator $\tilde{K}$, whose diagonal entries, $\sum_{k\neq i}r_{ki}$ have been produced automatically by the anticommutator of the off-diagonal jump operators  □.

**Note 4:** Lemma 2 is the formal statement of why the diagonal entries of $K_{\mu }$ must be excluded from the jump sum. The classical outflow is generated internally by the GKSL structure; as a result, reintroducing $K_{jj}$ as an additional jump rate can introduce a double count of the outflow in the coherence sector with the wrong sign; the consequences are elaborated in Appendix B.6.

### Appendix B.5. Contraction of Coherences (Lemma 3) and Stationarity (Lemma 4)

**Lemma 3.** 
*For* i≠j*, the dissipative part acts on the coherence* $\rho_{ij}$ *as pure exponential damping:*

$${\dot{\rho }}_{ij}\;{|}_{diss}=-1/2\;\left(a_{i}+a_{j}\right)\;\rho_{ij},\;\;a_{j}=\sum_{k\neq j}r_{kj}\geq 0,$$

*where* $a_{j}$ *is the total outflow rate from pillar* j*. Coherences are never amplified by the dissipative channels; the Hamiltonian part only rotates them*.

**Proof.** For i≠j, the sandwich term $E_{kl}\;\rho \;E_{lk}$ has off-diagonal matrix elements $\langle i\left|E_{kl}\;\rho \;E_{lk}\right|j\rangle =\delta_{ik}\delta_{jk}\rho_{ll}=0$, so the entire off-diagonal action comes from the anticommutator:

$$\langle i\left|\;\{E_{ll},\rho \}\;\right|j\rangle =\left(\delta_{il}+\delta_{jl}\right)\rho_{ij}.$$

Summing $1/2r_{kl}$ over all jumps $\left(k,l\right)$, k≠l, collects the total outflow rates of the two pillars involved, giving the stated decay with rate $1/2\left(a_{i}+a_{j}\right)\geq 0$. □

**Lemma 4 (For each channel).** 
*With symmetric coupling* $C_{ij}=C_{ji}$*, each classical generator satisfies detailed balance with respect to its own target distribution,*

$$k_{ij}^{\mu }\;\pi_{j}^{\mu }=\lambda_{\mu }\;C_{ij}\;\pi_{i}^{\mu }\;\pi_{j}^{\mu }=k_{ji}^{\mu }\;\pi_{i}^{\mu },$$

*Consequently,* $K_{\mu }\;\pi^{\mu }=0$ *and* $D_{\mu }\left(diag\pi^{\mu }\right)=0$*. As a result, one can argue that the attack dissipator is stationary exactly at the stream profile* s*, and the defense dissipator exactly at the audience prior* $p_{0}$.

**Proof.** The detailed-balance identity is immediate from the definition of the rates and the symmetry of C. Summing the balance identity over i≠j gives ${\left(K_{\mu }\pi^{\mu }\right)}_{j}=\sum_{i\neq j}k_{ji}^{\mu }\pi_{i}^{\mu }-\left(\sum_{i\neq j}k_{ij}^{\mu }\right)\pi_{j}^{\mu }=0$. For the dissipator, $diag\pi^{\mu }$ carries no coherences, so Lemma 3 gives no off-diagonal contribution, and Lemma 2 reduces the diagonal action to $K_{\mu }\pi^{\mu }=0$. □

**Note 5.** Lemma 4 elevates the attack-versus-defense interpretation from a model choice to a theorem. This means that each dissipative channel individually holds its own target fixed, and the composite dynamics relaxes to a $\left(\gamma ,q\right)$ weighted balance between the stream profile and the audience prior.

### Appendix B.6. Necessity of the Off-Diagonal Restriction

The restriction of the jump sum to i≠j in Appendix B.1 is necessary; including the diagonal entries of $K_{\mu }$ as jump rates yields a generator that is not completely positive.

Suppose the jump sum is extended to all pairs $\left(k,j\right)$, including k=j. The additional contribution is

$$\Delta \left(\rho \right)=\sum_{j}K_{jj}\left(E_{jj}\;\rho \;E_{jj}-1/2\left\{E_{jj},\rho \right\}\right),$$

with $K_{jj}=-a_{j}\leq 0$. On the diagonal, $\langle i\left|E_{jj}\rho E_{jj}\right|i\rangle =\delta_{ij}\rho_{ii}$ exactly cancels the anticommutator term, so Δ leaves all populations unchanged. On the coherences, however, the sandwich term contributes nothing ($\langle i\left|E_{ll}\rho E_{ll}\right|j\rangle =\delta_{il}\delta_{jl}\rho_{ij}=0$ for i≠j), while the anticommutator gives

$${\dot{\rho }}_{ij}\;{|}_{\Delta }=-1/2\left(K_{ii}+K_{jj}\right)\rho_{ij}=+1/2\left(a_{i}+a_{j}\right)\rho_{ij},$$

the exact negative of the damping in Lemma 3. Therefore, the corrupted dissipator relaxes populations toward $\pi^{\mu }$ while leaving the magnitudes of the coherences undamped. This violates the positive-semidefiniteness of ρ, which requires the Schwarz inequality

$${\left|\rho_{ij}\right|}^{2}\leq \rho_{ii}\;\rho_{jj}$$

This must always hold true, hence, starting from a state with nonzero coherence, the right-hand side decays toward $\pi_{i}^{\mu }\pi_{j}^{\mu }$, scale values while the left-hand side persists, and the inequality eventually fails, at which point $\rho \left(t\right)$ acquires a negative eigenvalue and ceases to be a density matrix.

The failure is also visible directly at the level of the GKSL theorem. In the operator basis $\left\{E_{kj}\right\}$ extended to include the diagonal units, the Kossakowski matrix of the corrupted generator is $diag\left(r_{kj};\;K_{jj}\right)$, which contains the strictly negative entries $K_{jj}=-a_{j}$ whenever any outflow rate is nonzero. Since the GKSL characterization is an equivalence, no rewriting of the corrupted generator can ensure complete positivity. This is because the negative eigenvalues of its Kossakowski matrix are basis-independent obstructions. Numerically, the defect was confirmed by a Choi Matrix test: a map Φ is completely positive if and only if its Choi matrix $J\left[\Phi \right]=\sum_{a,b}E_{ab}⊗\Phi \left(E_{ab}\right)$ is positive semidefinite. In the context of this model, the corrupted generator producMes minimum Choi eigenvalues of order $-10^{-3}$ at $dt=10^{-3}$ and drives an initially coherent state to a minimum population eigenvalue of approximately −0.10; on the other hand, the generator discussed in Appendix B.1 passes the same test to within numerical tolerance for all tested γ, and q.

To sum up, the generator $L_{total}$, which is built for this model in Equation (13), is a single GKSL generator with effective Hamiltonian $\tilde{H}=\gamma qH$ and effective jump rates $r_{ij}=\gamma \left(1-q\right)k_{ij}^{attack}+\left(1-\gamma \right)k_{ij}^{defense}$. The characteristics of this generator can be listed as follows: (1) It generates a completely positive, trace-preserving quantum dynamical semigroup for every $\left(\gamma ,q\right)\in {\left[0,1\right]}^{2}$ shown in Theorem 1; (2) it preserves normalization, shown in Lemma 1; (3) its population sector reproduces exactly the classical Kolmogorov dynamics of the weighted generator $\tilde{K}$, shown in Lemma 2; (4) its dissipative channels damp coherences, shown in Lemma 3; (5) each channel is individually stationary at its own target distribution, so that the composite dynamics realizes a mathematically defined competition between the information stream and the audience prior, shown in Lemma 4. The restriction of the jump operators to the off-diagonal matrix units is necessary for these conclusions, shown in Appendix B.6.

## Appendix C. Detection and Correction of a Complete-Positivity Defect in the Numerical Implementation

Appendix B proves that the generator $L_{total}$ in Equation (13) is a valid GKSL, a.k.a, Lindblad equation, generator when constructed as specified to capture separate attack and defense dynamics. This appendix elucidates what happens when one step of that construction is incorrect, how the error can be recognized, and how the correct implementation is included in the simulation.

### Appendix C.1. The Complete Positivity Defect

The dissipator of each classical channel is assembled by converting the Kolmogorov generator $K_{\mu }$ into a GKSL dissipation superoperator. This is being done by looping over matrix entries and attaching a jump operator $E_{kj}=\left|k\rangle \langle j\right|$ with rate $K_{kj}$ to each of them. The GKSL-valid construction as shown in Appendix B.1 uses only the off-diagonal entries $k_{kj}^{\mu }\geq 0$, k≠j, as jump rates:

$$D_{\mu }\left(\rho \right)=\sum_{k\neq j}k_{kj}^{\mu }\left(E_{kj}\;\rho \;E_{jk}-1/2\left\{E_{jj},\;\rho \right\}\right).$$

In this context, a defective implementation can be a loop over all of the index pairs $\left(k,j\right)$, including k=j. Since the diagonal entries of a Kolmogorov generator are the negative total outflow rates, $K_{jj}=-\sum_{i\neq j}k_{ij}^{\mu }\leq 0$, the loop includes an additional jump operator $E_{jj}=\left|j\rangle \langle j\right|$ carrying a negative rate. This is a problem because a negative jump rate is what the GKSL theorem does not accept; the reason is that the Kossakowski matrix of the resulting generator gains negative diagonal entries, and validity cannot be restored as shown in Appendix B.6. If this is not monitored or fixed as in [48], the generator produced by a possibly defective loop and hence the model evolution would be represented by a generator of a completely positive evolution.

Conceptually, the error can be described as follows. The diagonal of $K_{\mu }$ is the necessary part of the classical generator; it is what makes the columns sum to zero, and the populations conserve probability. So, carrying it into the quantum dissipator can be seen as completeness rather than error. The resolution is Lemma 2 of Appendix B shows that in the GKSL structure the diagonal outflow is generated automatically by the anticommutator of the off-diagonal jumps, via the operator identity

$$\sum_{k\neq j}k_{kj}^{\mu }\;E_{kj}^{†}E_{kj}=\left(\sum_{k\neq j}k_{kj}^{\mu }\right)E_{jj}=-K_{jj}\;E_{jj}.$$

Adding the diagonal entry as a jump does not complete the process of checking/monitoring consistency of the classical component of the model. The reason for not completing is that the anti-commutator generated by the off-diagonal jumps supplies the outflow term $-a_{j}p_{j}$, where $a_{j}=\sum_{k\neq j}k_{kj}^{\mu }$ is the total outflow rate from MFT pillar j, in the population equation, the damping $-\frac{1}{2}\left(a_{i}+a_{j}\right)\rho_{ij}$ in the coherence equation, so every rate in $K_{\mu }$ has already acted on both sectors. The extra diagonal jump $E_{ij}$ with rate $K_{jj}=-a_{j}$ then enters the simulation/calculation a second time. This second entry in the foundation probabilities (population is the common term in the open system discussion) generates two contributions which cancel identically, leaving no trace, and introduce $+\frac{1}{2}\left(a_{i}+a_{j}\right)\rho_{ij}$ to the coherence. The result is that a dissipator in this calculation has correct probability entries, but evolves with a non-decaying coherence. The reason for non-decay is that since classical outflow is double-counted in the off-diagonal elements, the copies annihilate each other because of the sign flips.

### Appendix C.2. What the Defect Affected & Why It Was Invisible

The extra diagonal-jump term is

$$\Delta \left(\rho \right)=\sum_{j}K_{jj}\left(E_{jj}\;\rho \;E_{jj}-1/2\left\{E_{jj},\;\rho \right\}\right),$$

and its action is separated by both diagonal (population, foundation probabilities) and non-diagonal (coherence) elements.

The foundation probabilities, also known as populations, the diagonal elements of, ρ are identically zero. On the diagonal, $\langle i\left|E_{jj}\rho E_{jj}\right|i\rangle =\delta_{ij}\rho_{ii}$ cancels the anticommutator contribution. All the foundation probabilities $p_{i}\left(t\right)$, their stationary values, total variation distance, net shift, and dominant-pillar statistics, are calculated identically by the defective and corrected completions.

For the coherence, the off-diagonal elements of ρ, the sandwich term does not contribute to off-diagonal, while the anticommutator contributes as follows.

$${\dot{\rho }}_{ij}\;{|}_{\Delta }=-1/2\left(K_{ii}+K_{jj}\right)\rho_{ij}=+1/2\left(a_{i}+a_{j}\right)\rho_{ij},\;\;a_{j}=\sum_{k\neq j}k_{kj}^{\mu },$$

where is the negative of the legitimate coherence damping shown in Appendix B, Lemma 3. The defective dissipator relaxes populations correctly while leaving coherence magnitudes undamped. In this situation, an anti-decoherence term eventually drives $\rho \left(t\right)$ to violate the Schwarz inequality ${\left|\rho_{ij}\right|}^{2}\leq \rho_{ii}\rho_{jj}$ and gain negative eigenvalues. In numerical experiments at the parameters of this study, an initially coherent state evolved under the defective generator could reach a minimum eigenvalue of approximately −0.10; this means that the matrix is no longer a density matrix.

This sector separation (probabilities vs. coherence) explains why the defect survived the standard verification checklist. For example, (1) trace preservation held, in which the defective generator was still trace-preserving and Hermiticity-preserving, so normalization checks (Trρ=1 at every step) passed; (2) the classical limit held, which means at q=0 with a diagonal initial state, the dynamics reduces to the classical master equation and never generates coherence, so the broken sector is never exercised. Trajectory tests initialized at probability distributions, thus, passed identically; (3) stationarity check, which can be done via the null-space check, $K_{\mu }\pi^{\mu }=0$ operates on the classical generator itself, not on the dissipator built from it, and is unaffected. As a possible solution, the trajectory-based tests certify only the states they visit. This solution does not ensure because complete positivity is a statement about all inputs simultaneously, including states carrying coherence, so no finite checklist of trajectory checks from classical initial states can establish it. A state-independent criterion is required such that this criterion should explicate the coherence component with a coherent input; this is further discussed in the following sections.

An alternative solution, used in [48] was considered as well. In that construction, the jump rates are drawn not from the generator K itself but from the scaled transition matrix $G=T\left(h\right)/h$, where $T\left(h\right)=e^{Kh}$ and h are small time steps. Since $T\left(h\right)$ is a stochastic matrix, every entry in the matrix is non-negative, so all jump rates are admissible and the resulting dissipator is completely positive by construction, which is a solution. However, by altering the dynamics rather than by removing the defect, and the alteration falls on the sectors (probabilities and coherence) this modeling study measures.

Two structural dynamics govern the construction. First, the population (probabilities) sector evolves under $\left(T\left(h\right)-I\right)/h$ rather than under K. This evolution parameter is a first-order approximation whose relaxation spectrum is near −1/h. Second, because the columns of $T\left(h\right)$ sum to unity, the diagonal entries $T_{jj}/h$ act as pure-dephasing jumps. Their combined damping of every coherence is 1/h, independent of the model rates. The strength of the quantum-classical interface is thus set by a discrete parameter instead of the model parameters $\left(\gamma ,q\right)$.

Through this approach [48], negativity can be prevented; however, it is a suppression rather than a correction. The reason is that the anti-damped coherences that would violate positivity are crushed by the 1/h dephasing before they can do so. For a two-state model application, this may be an acceptable solution; however, for this work, it is not a fit because the suppressed quantities, which are coherent oscillation, superposition ambiguity, and the coherence on which the non-selective elicitation measurement, in the simulation model, acts, are the investigated quantities.

The approach adopted here separates the two concerns. The defect is removed at its source, and no need to suppress the corrected dissipator implements the generator specified by the theory, with no discretization parameter entering the evolution dynamics. Validity of the implemented solution is then monitored rather than enforced. This means that the Choi certification of Appendix C.4 verifies complete positivity of the generator without perturbing the dynamics. The distinction is between securing positivity by suppressing the state and certifying positivity while leaving the state free to evolve as the model requires.

### Appendix C.3. The Implementation of the Correction

The correction is the restriction of the jump loop to off-diagonal entries, which is as follows

**for** j = 1:dim **for** k = 1:dim **if** k == j, **continue**; **end** *% off-diagonal jumps only -> CP-valid* Lop = sparse((x==k)*(x==j)′); LdL = Lop′*Lop; D = D + K(k,j)*(kron(Lop,Lop) − 0.5*(kron(LdL,ID) + kron(ID,LdL))); **end** **end**

The implementation of this fix does not change how the dynamics are built and calculated in the simulation. The classical generators $K_{attack}$ and $K_{defense}$ retain their diagonal entries; those are required for the classical dynamics and for the stationarity check. The population dynamics are unchanged by construction as discussed in Appendix C.2; the correction is not a re-tuning of the model, rather, it restores the coherence sector to the behavior the theory specifies while leaving the classical embedding as it is. The implemented dissipator coincides with the object analyzed in Appendix C, so Theorem 1 and Lemmas 1–4 apply to the calculation as the simulation runs.

### Appendix C.4. The Choi Matrix Criterion and Simulation Verification

The corrected implementation verifies its own generator. The verification capitalizes on the Choi–Jamiołkowski isomorphism. For a linear map Φ on dim×dim matrices, the Choi matrix is defined as [72,73]:

$$J\left[\Phi \right]=\sum_{a,b=1}^{d}E_{ab}⊗\Phi \left(E_{ab}\right),$$

a ${dim}^{2}\times {dim}^{2}$ where dim=5, the Choi matrix is a 25×25 matrix in the model. Choi’s theorem states that Φ is completely positive if and only if $J\left[\Phi \right]\geq 0$. The theorem translates a generalized quantified property of positivity of the output for all inputs, including the entangled extensions. The exception would be when J[Φ] becomes the output of Φ acting on part of a maximally entangled state [72,73]. One eigenvalue decomposition of one matrix can settle complete positivity for all inputs at the same time. Trace preservation appears as the normalization $TrJ\left[\Phi \right]=dim$, and the defective generator has to satisfy this trace condition, which is why a normalization-based test could not detect this defect.

Validity of a generator means complete positivity of the generated maps $e^{tL}$ for all t≥0; The validity check is applied to the propagator at an infinitesimal probe time. A CP validity check in the simulation forms $\Phi_{\delta }=e^{L\;\delta }$ with $\delta =10^{-3}$, that assembles $J\left[\Phi_{\delta }\right]$, and tests the minimum eigenvalue against a small negative tolerance. This delivers ontologically robust monitoring steps, which are as follows. (1) For small δ, $J\left[e^{L\delta }\right]\approx J\left[id\right]+\delta \;J\left[L\right]$, a non-GKSL generator produces a negative Choi eigenvalue scaling linearly in δ times the negative eigenvalue of its Kossakowski matrix [72,73]. Since the defect cannot be hidden at small δ, any CP failures are noticed at first order. (2) If $e^{L\delta }$ is completely positive, so is $e^{Ln\delta }={\left(e^{L\delta }\right)}^{n}$ for every n. Consequently, the compositions of completely positive maps become positive. The check is computed once per audience profile, immediately after $L_{total}$ is assembled, and issues a warning if the criterion ever fails.

### Appendix C.5. Verification Results for the Implemented Choi Test

Table A2 shows the minimum Choi eigenvalue of $e^{L_{total}\delta }$, $\delta =10^{-3}$, for the defective and corrected constructions with the parameter values of this study, which are hashtags in the information stream, $\lambda_{attack}=5,{\;\lambda }_{defense}=5$, $V_{0}=500$, and the block coupling v=0.3.

**Table A2:** Choi eigenvalue monitoring for the shift between control and treatment conditions analysis.

γ	q	Construction	Minimum Choi Eigenvalue	CP
0.5	1.0	Corrected, off-diagonal	$+2.1\times 10^{-7}$	Pass
0.5	1.0	Defective	$-1.3\times 10^{-3}$	Fail
0.5	0.5	Corrected	$+3.1\times 10^{-7}$	Pass
0.5	0.5	Defective	$-2.1\times 10^{-3}$	Fail
0.5	0.0	Corrected	$+4.0\times 10^{-7}$	Pass
0.5	0.0	Defective	$-2.8\times 10^{-3}$	Fail
1.0	1.0	either	$\approx -1\times 10^{-15}$	Pass

The first information to extract from this table is the defective construction that fails at every parameter point when a dissipator enters the calculation of the dynamics, with negative eigenvalues in the order of $10^{-3}$ scale that can be predicted (in the simulation) by the first-order analysis; for example, the second of γ=0.5, q=0.5 cases in Table A2. The second piece of information is that at γ=1,q=1 both constructions coincide and pass. This point in this case is that it is pure Hamiltonian evolution; no dissipator enters, and the residual ~−10^−15^ reflects a unitary channel on the boundary of the completely positive set, which is a rank-one Choi matrix, not a near-failure. To identify this type of boundary condition, a runtime check compares against a tolerance rather than against a zero threshold. The third piece of information is that a corrected construction passes with a positive margin that grows linearly with the probe time interval; the signature of a generator stays inside this valid set.

The Choi matrix monitoring of CP in fact maintains a continuous diagnostic with the corrected generator, and for γ=0.5, q=1 values in Table A2, one can confirm the restored behavior: the minimum eigenvalue of $\rho \left(t\right)$ remains nonnegative within the implemented tolerance threshold over the full simulated horizon. This mean that ρ is always a density matrix, the trace is conserved to twelve decimal places. Moreover, this Choi test ensures that the $L_{total}$ coherence decays monotonically apart from Hamiltonian-driven oscillation, rather than persisting undamped; and the semigroup composition property $e^{2tL}={\left(e^{tL}\right)}^{2}$ holds at the $\sim 10^{-16}$ for all simulation conditions.

The sector separation, probabilities, and coherence, as discussed in Appendix C.2, introduce a demarcation line for the quantities that can and cannot be affected by the implemented correction. All of the parameters that are computed from the diagonal terms of the ρ under measurement-free evolution, such as foundation probability trajectories, total variation distance to the stream (TVD), signed net shift, and dominant-pillar identification. On the other hand, all coherence-dependent quantities can be affected. In fact, the updated probability figures in the version of the study indicate this. Under the defective construction, the magnitude of the oscillation (coherence) was systematically inflated; the consequence of this is that (1) purity (not MFT, density matrix purity) and von Neumann entropy traces can be biased, and (2) any effect of the non-selective elicitation measurement can be affected in the simulation. This is because the operation that generates this outcome operates by removing coherences, and hence, its effect can scale with the coherence present at the time of the act of measurement in the simulation model. Therefore, all of the elicitation results explicated in this study have been reproduced with the corrected implementation that is based on criterion of Appendix C.4.

## Appendix D. Sensitivity of the Reported Ordering to the Hamiltonian Constants c and *V_0_*

The Hamiltonian that is shown in Equation (5) consists of two constants. These are the coupling strength c=35 and the stream-potential depth $V_{0}=500$, adopted from an earlier open-system quantum walk study [48]. As discussed in Section 4, these are modeling constants rather than empirically estimated parameter. The reason for these modeling constants selection is that the time unit of the model is arbitrary, the magnitudes of these parameter values do not carry any independent meaning. In the model implementation, only the relative scale is significant. This appendix provides a quantitative discussion on whether the paper’s comparative conclusions depend on the chosen values.

### Appendix D.1. Design of the Sensitivity

In this sensitivity analysis, each constant was varied over a factor of four in both directions around their nominal values that are used in this study. This results in producing a 5×5 grid including a sixteen-fold range per parameter which are as follows.

c∈{8.75, 17.5, 35, 70, 140}

and

$$V_{0}\in \left\{125,\;250,\;500,\;1000,\;2000\right\}.$$

At each of the 25 grid points, $L_{total}$ was calculated as described in Section 4 and Appendix B, with the following values:

- MFT block coupling, ν=0.3;
- $\lambda_{attack}=\lambda_{defense}=5$;
- γ=0.5, q=1

All other elements of the model, such as the streams’ profiles, the audience priors, and the classical channels, do not include c or $V_{0}$, and they are held constant.

The comparison metric is the total variation distance (TVD) of the audience’s foundation probabilities to the stream profile over an averaging time window. This averaging time window covers the pre-equilibrium phase of the evolution, during which the Hamiltonian constants shape the dynamics; at later times the state is dominated by the γ balanced dissipative stationary point and only weakly sensitive to c and $V_{0}$.

### Appendix D.2. Results of the Sensitivity Analysis

Figure A3 shows the time-averaged distance for the conservative audience (left), the progressive audience (center), and their difference (right), with the nominal operating point marked in a red box. There are three points to elaborate.

First, the qualitative ordering that carries the paper’s comparative claims is preserved at all of the grid points. For example, the conservative audience remains closer to the stream than the progressive audience in combinations (25 of 25 comparison cases), with the gap ranging from −0.134 to −0.299. The reported asymmetry between audiences is therefore not a property of the particular constants c=35, $V_{0}=500$.

Second, the randomness of the absolute scale is confirmed by simulation experiment. Along the main diagonal of the grid the ratio $V_{0}/c$ is held fixed while both constants are simultaneously rescaled by a factor of sixteen. In this situation, the metric is unchanged along this diagonal (conservative: 0.234, 0.221, 0.228, 0.235, 0.229; progressive: 0.422, 0.413, 0.416, 0.419, 0.416). The purpose joint rescaling of the Hamiltonian is to rescale only the time axis; thus, the outcomes of the sensitivity analysis depend on the ratio of coupling to potential.

Third, the residual variation across the grid is physically interpretable. In the potential-dominated, small c, large $V_{0}$ area, the progressive audience remains near its prior (distance ≈0.50, the baseline). Another way of interpreting this is that a strong moral bias combined with weak inter-foundation coupling, ν, localizes the state. This is because large diagonal energy disparities suppress possible coherent transfers between foundations. On the other hand, in the coupling-dominated large c, small $V_{0}$ situations, both audiences move/mix toward the stream more effectively. The phenomenon that is examined in this study is in the region where coherent mixing and the streams’ moral bias compete. Therefore, as shown in this Appendix D, the nominal values place the dynamics between these regimes.

### Appendix D.3. Summary

The sensitivity analysis shows the analysis of fifty generators (25 grid points × 2 audience profiles) with parameter combinations not explicated in the main analysis. Complete positivity (CP) of every generator was verified by the Choi criterion, which is shown in Appendix C. The minimum Choi eigenvalue across the entire simulation was $+8.1\times 10^{-8}$, a strictly positive margin confirming that all dynamics underlying Figure A3 are completely positive and trace preserving. This computation is deterministic; matrix exponentials of fixed operators were used, with no stochastic elements.
